# A dual druggable genome-wide siRNA and compound library screening approach identifies modulators of parkin recruitment to mitochondria

**DOI:** 10.1074/jbc.RA119.009699

**Published:** 2020-01-07

**Authors:** Helen L. Scott, Nicola Buckner, Francesc Fernandez-Albert, Elisa Pedone, Lorena Postiglione, Gongyu Shi, Nicholas Allen, Liang-Fong Wong, Lorenzo Magini, Lucia Marucci, Gregory A. O'Sullivan, Sarah Cole, Justin Powell, Peter Maycox, James B. Uney

**Affiliations:** ‡ Bristol Medical School, University of Bristol, Bristol BS8 1TD, United Kingdom; § Takeda Cambridge Ltd., Cambridge Science Park, Cambridge CB4 0PZ, United Kingdom; ¶ Department of Engineering and Mathematics, University of Bristol, Bristol BS8 1TD, United Kingdom; ‖ School of Cellular and Molecular Medicine, University of Bristol, Bristol BS8 1TD, United Kingdom; ** School of Biosciences, Cardiff University, Cardiff CF10 3AX, United Kingdom; ‡‡ BrisSynBio, Bristol BS8 1QU, United Kingdom; §§ Takeda Ventures, Inc., 61 Aldwych, London WC2B 4A, United Kingdom

**Keywords:** mitochondria, parkin, ubiquitin, Parkinson disease, mitophagy, kenpaullone, genome screen, neurodegeneration, ubiquitin proteasome system (UPS)

## Abstract

Genetic and biochemical evidence points to an association between mitochondrial dysfunction and Parkinson's disease (PD). PD-associated mutations in several genes have been identified and include those encoding PTEN-induced putative kinase 1 (PINK1) and parkin. To identify genes, pathways, and pharmacological targets that modulate the clearance of damaged or old mitochondria (mitophagy), here we developed a high-content imaging-based assay of parkin recruitment to mitochondria and screened both a druggable genome-wide siRNA library and a small neuroactive compound library. We used a multiparameter principal component analysis and an unbiased parameter-agnostic machine-learning approach to analyze the siRNA-based screening data. The hits identified in this analysis included specific genes of the ubiquitin proteasome system, and inhibition of ubiquitin-conjugating enzyme 2 N (UBE2N) with a specific antagonist, Bay 11-7082, indicated that UBE2N modulates parkin recruitment and downstream events in the mitophagy pathway. Screening of the compound library identified kenpaullone, an inhibitor of cyclin-dependent kinases and glycogen synthase kinase 3, as a modulator of parkin recruitment. Validation studies revealed that kenpaullone augments the mitochondrial network and protects against the complex I inhibitor MPP+. Finally, we used a microfluidics platform to assess the timing of parkin recruitment to depolarized mitochondria and its modulation by kenpaullone in real time and with single-cell resolution. We demonstrate that the high-content imaging-based assay presented here is suitable for both genetic and pharmacological screening approaches, and we also provide evidence that pharmacological compounds modulate PINK1-dependent parkin recruitment.

## Introduction

Parkinson's disease (PD)^2^ is the second most common neurodegenerative disease and is characterized by the degeneration of midbrain dopaminergic (DA) neurons. Historic biochemical research suggested that interlinked pathways, governing free-radical production, iron metabolism, and mitochondrial function were altered in PD. Genetic and molecular evidence has since confirmed an association between mitochondrial dysfunction and PD (1–3). In rare familial cases of PD, causal mutations in a number of coding genes have been identified and include PTEN-induced putative kinase 1 (*PINK1*), parkin (*PRKN*), α-synuclein (*SNCA*), leucine-rich repeat kinase 2 (*LRRK2*), ATPase 13A2 (*ATP13A2*), *VPS35* (*PARK17*), and Parkinsonism-associated deglycase (*PARK7*)/DJ-1 (4). Functional dissection of these genes found that systems governing mitochondrial quality control, the ubiquitin proteasome system, and protein trafficking and clearance through the autophagosomal-lysosomal network are involved in the etiology of PD (5). Mitochondrial dysfunction, possibly due to mutations in mitochondrial DNA, has also been shown to increase the risk of developing PD and other late onset human diseases, such as multiple sclerosis and Rett syndrome (6–8). In addition, exposure to 1-methyl-4-phenyl-1,2,3,6-tetrahydropyridine and its active metabolite 1-methyl-4-phenylpyridinium (MPP+) inhibits mitochondrial respiratory complex I in DA neurons and leads to a form of Parkinsonism (9). Furthermore, the risk of developing PD is increased by exposure to pesticides, such as rotenone, that also affect mitochondrial respiratory complex I (10).

Mitochondria are exceptionally dynamic organelles; they undergo continuous rounds of biogenesis, fusion, fission, and removal to ensure that the cell has a population capable of meeting its metabolic demands. A key event in this life cycle is the decision to clear old/damaged mitochondria by the selective use of the macroautophagic process termed mitophagy. In healthy mitochondria, which maintain a normal membrane potential, PINK1 is recruited to and rapidly transported across the outer mitochondrial membrane (OMM) to the inter membrane space, where it is cleaved by proteases, including mitochondrial processing peptidase and presenilin-associated rhomboid-like (11, 12). When mitochondria become depolarized (*e.g.* due to oxidative phosphorylation inhibition, depolarizing agents, mitochondrial DNA mutations, or disease) PINK1 cannot be imported, and it accumulates on the OMM (13, 14). Mitochondrial proteins are constitutively ubiquitinated by E3 ligases such as MITOL (membrane-associated ring-CH-type finger 5; MARCH5). Accumulated PINK1 phosphorylates the ubiquitin chains and triggers parkin recruitment (15). Subsequently, parkin is phosphorylated by PINK1, resulting in the activation of its E3 ligase activity (16–19). Activated parkin leads to further ubiquitination of mitochondrial proteins in a positive feedback cycle and so signals the mitochondrion for autophagic degradation via recruitment of autophagy adapters, such as sequestosome 1 (SQSTM1/p62) and microtubule-associated protein 1 light chain 3 α/microtubule-associated protein 1 light chain 3 β 2 (MAP1LC3A/B2). We employed a phenotypic assay based on parkin recruitment to identify genes and compounds that modulate this pathway (20–23). To focus on disease-relevant targets, we screened a druggable-genome siRNA library (targeting 7500 genes) and used multiparametric analysis and a parameter-agnostic machine-learning approach to maximize the identification of hits and key nodes that drive parkin recruitment. Second, we screened a library of neuroactive compounds to identify modulators of mitophagy and thus potential therapeutic options.

We validated hits from both screens by investigating effects on (i) the downstream ubiquitination and degradation of OMM proteins; (ii) the mitochondrial network; and (iii) the ability to protect against the mitochondrial toxin MPP+. Furthermore, we established a microfluidics/microscopy-based assay to monitor parkin recruitment in real time. Together, we show how combining multiple screening approaches can aid the discovery of targetable master regulators of mitophagy and help the identification of pharmacological strategies that promote mitochondrial function and present new therapeutic options for PD.

## Results

**Figure 1.**
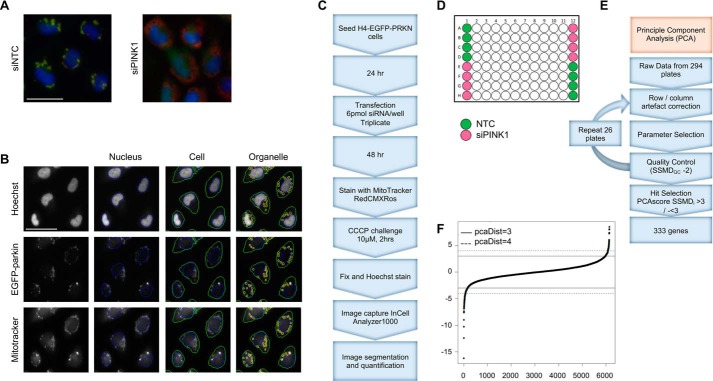
**Screening of the druggable genome library.** *A*, overlay of example images captured by the InCell Analyzer of H4-EGFP-PRKN cells stained with MitoTracker Red and Hoechst following 2-h CCCP treatment. *B*, example of images captured by the InCell Analyzer and the segmentation of nuclei (*blue*), cells (*green*), and EGFP-PRKN puncta (*Organelles*) (*yellow*). *C*, schematic of mitophagy assay, image capture, and quantification workflow. *D*, 96-well plate layout showing the position of NTC and siPINK1 wells. *E*, PCA analysis pipeline. *F*, PCA score data showing thresholds of >3 and <−3. *Scale bars*, 50 μm.

We established an assay of parkin recruitment that was suitable for high-throughput screening. EGFP-tagged parkin (EGFP-PRKN) was used to visualize parkin translocation to the mitochondria following treatment with the protonophore carbonyl cyanide 3-chlorophenylhydrazone (CCCP) to dissipate Ψ_m_ (Fig. S1, *A* and *B*). In addition, MitoTracker Red and EGFP-PRKN puncta showed a nearly complete colocalization following treatment with CCCP (Fig. S1*A*). Cells transfected with a nontargeting control (NTC) siRNA showed robust recruitment of parkin to the mitochondria and peri-nuclear clustering of parkin-tagged mitochondria, whereas siPINK1-transfected cells showed no recruitment of EGFP-PRKN, and mitochondria remained distributed throughout the cytoplasm (Fig. 1*A*). The images were segmented to identify and quantify nuclei (*Nucleus*), cell bodies (*Cell*), and parkin puncta (*Organelle*) (see “Experimental procedures” for details; Fig. 1*B*). Initial experiments in which EGFP-PRKN and siRNA were co-transfected showed heterogeneity in the level of EGFP-PRKN expression, resulting in poor reproducibility and suboptimal assay windows between positive (siPINK1) and negative (NTC) controls (Fig. S2, *A* and *B*), and a stable EGFP-PRKN cell line was therefore made (Fig. S3). The concentration and duration of CCCP treatment was then optimized to ensure a robust assay window allowing detection of both positive and negative modulators of mitophagy (Fig. S4). A primary screen using the Dharmacon OnTarget Plus druggable genome (7514 genes) Smartpool (4 siRNAs/gene) siRNA library was carried out using our H4-EGFP-PRKN cells (Fig. 1*C*) together with NTC (negative control) and PINK1-targeting (siPINK1) (positive control) siRNAs included on all plates (Fig. 1*D*). All plates were transfected and assayed in triplicate. 48 h post-transfection, cells were stained with MitoTracker Red CMXRos (100 nm, 1 h) and treated with CCCP (10 μm, 2 h) prior to being fixed, and the nuclei were labeled with Hoechst.

After quality control, 6138 genes were carried forward to the hit selection stage, where we used the principal components analysis (PCA) workflow (see “Experimental procedures”) to score the genes (Fig. 1*E*). The distribution of the values follows a cumulative normal distribution (Fig. 1*F*). Setting a PCA score cut-off of either >3 or <−3, a collection of 333 genes were identified as hits within the primary screen (File S1). The hits of this list showed a statistically significant enrichment for previously reported parkin interactors (24) (see “Parkin interactor enrichment”) with a *p* value of 0.0058.

**Figure 2.**
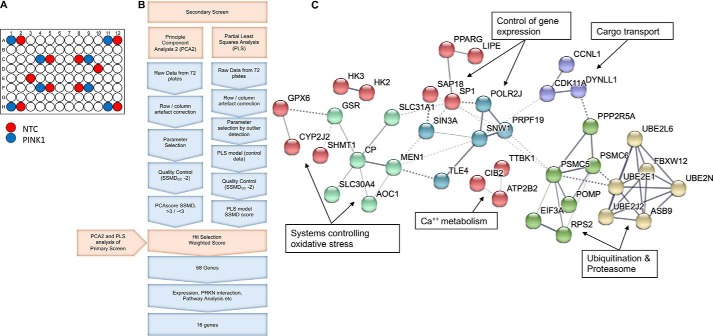
**Secondary screen analysis pipeline and results.** *A*, 96-well plate layout showing the position of NTC and siPINK1 wells. *B*, *schematic* of the analysis pipeline for the secondary screen. *C*, STRING analysis showing the interactions (known and predicted) between the hit genes identified in the secondary PRKN screen and the cellular pathways controlled by these gene clusters. *Width* of *edge* denotes confidence of interaction.

To validate the results of the primary screen and control for off-target effects, the top 300 hits from the primary screen were carried forward to a secondary screen (Fig. 2). For this, we had a custom library of Ambion Silencer Select siRNAs synthesized consisting of three individual siRNAs per gene (File S2). H4-EGFP-PRKN cells were assayed in the same manner as for the primary screen with the exception that the siRNA concentration of the single siRNAs was reduced to 0.25 pmol/well, and all plates were run with *n* = 6. In addition, the positioning of NTC and siPINK1 controls were adjusted to better control for row and column effects (Fig. 2*A*). Analysis of the data using the PCA score (for the three previously identified key parameters) revealed that the PRKN puncta/cell intensity parameter poorly separated the NTC from PINK1 controls. As a PCA score value based on two parameters is suboptimal, a parameter-agnostic machine-learning approach (partial least squares; PLS) was taken to analyze the data to identify phenotypic hits (Fig. 2*B*) (for a full description, see “PLS workflow”). Analysis of the data based on this PLS score revealed that a significantly strong effect (SSMD > 1.3 or SSMD < −1.3) could only be found for all three secondary screen siRNAs in the same direction as the significant change for the smart pool in the primary screen for three genes, but there were 22 gene hits where this effect was found in two of three siRNAs and 55 genes where this effect was found in one of three siRNAs (see last column in File S3). To identify the most significant and robust hits, the gene SSMD values from both the primary and secondary screens were combined in the form of a weighted score (File S3). As the different quality numbers, corresponding to the different studies, are not comparable with each other, this field contains the mean value of all of the normalized PCA and PLS measures (*i.e.* normalized to their respective absolute maximum value). This is only possible as both the SSMD and the PCA measures correspond to their actual effect measures (and are not just significance measures as in the case of *p* values). As the weighted score is computed through the mean, genes showing opposite behaviors were penalized across all of the different studies and methods. To select genes for further analysis, we carried forward the top 10% positive and 10% negative regulating genes based on this weighted score (corresponding to a weighted score of >0.637 or <−0.6880), which resulted in a total of 58 genes (top 29 and bottom 29 genes from the summary table in File S3). Gene ontology analysis of these 58 genes revealed that there was an enrichment for “cellular metabolic process” and “organic substance metabolic process,” whereas PANTHER pathway analysis revealed enrichment for “ubiquitin proteasome pathway” (25). Similarly, STRING database analysis (Fig. 2*C*) revealed clusters of genes involved in ubiquitination, proteasomal degradation, control of oxidative stress (*e.g.* genes involved in GSH biosynthesis, copper, and zinc metabolism), calcium metabolism, cargo/organelle transport, and control of gene expression (DNA binding/transcriptional regulation) (Fig. 2*C*) (26). We selected 16 genes (File S4) for further validation according to a number of criteria, including (i) having a known gene expression profile in H4 cells (see “Gene expression in H4 cells”); (ii) evidence of parkin binding (24); (iii) general links to mitochondrial function and/or neurodegeneration; and (iv) occurrence of multiple hit genes within a functional pathway as identified by STRING, gene ontology, and PANTHER pathway analyses.

We used qPCR to determine whether the siRNAs used in the secondary screen resulted in an effective knockdown of the corresponding target (Fig. S5). For 11 of 16 genes (*ATP2B2, CIB2, DPYSL2, GSTK1, HK2, PPARG, SLC31A1, UBE2E1, UBE2J2, UBE2L6,* and *UBE2N*), there were at least two siRNAs that mediated a significant reduction in the target mRNA compared with the NTC RNA. For two genes (GSR and TTBK1), it transpired that only one siRNA sequence had been provided in the library, and for four genes (*CIB2, SLC31A1, UBE2E1,* and *UBE2J2*), only two sequences had been provided. GPX6 failed to amplify under the reaction conditions used. The siRNAs mediating the most robust knockdown of each target were used in subsequent validation studies.

**Figure 3.**
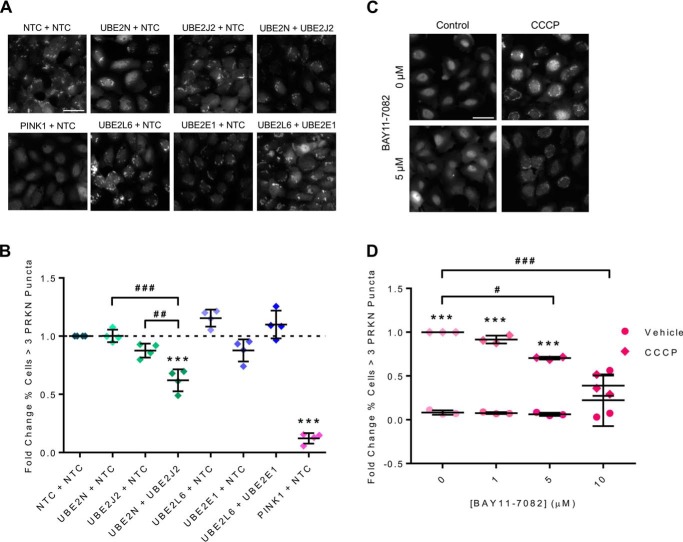
**UBE2N and UBE2J2 are positive modulators of PRKN recruitment.** *A*, representative images of PRKN recruitment in H4-EGFP-PRKN cells transfected with the indicated combinations of siRNAs and treated with CCCP (10 μm, 2 h). *B*, quantification of PRKN recruitment as displayed in *A*; there was a significant effect of siRNA (*F*_(7,24)_ = 77.84, *p* < 0.0001), and post hoc tests revealed that simultaneous knockdown of UBE2N and UBE2J2 enhanced the impairment of PRKN recruitment compared with knockdown of the individual genes (*p* < 0.0001) (*n* = 4). *C*, representative images of H4-EGFP-PRKN cells treated with the UBE2N inhibitor Bay 11-7082 and CCCP (10 μm, 2 h) as indicated. *D*, quantification of PRKN recruitment, as shown in *C*, identified a dose-dependent inhibition of CCCP-induced PRKN recruitment by Bay 11-7082 (*F*_Bay(3,16)_ = 5.309, *p* < 0.01) (*n* = 3). *Scale bar*, 50 μm. *Lines*, mean ± S.D. (*error bars*). *, significant difference from NTC/vehicle control; #, significant difference between *highlighted pair*; #, *p* < 0.05; ##, *p* < 0.01; ***/###, *p* < 0.001.

Due to the functional clustering of many of the hits, we decided to investigate whether targeting multiple genes (within a cluster) at the same time increased the observed effect on parkin recruitment. One of the clusters included members of the E2 ubiquitin-conjugating enzymes (UBE2s) whose activity is required for the activation of E3 ubiquitin ligases (UBE3s) like parkin. Of the four UBE2s identified, UBE2N and ubiquitin-conjugating enzyme E2 J2 (UBE2J2) were shown to be positive modulators of parkin recruitment (their knockdown reduced parkin recruitment in the screen), and ubiquitin-conjugating enzyme E2 L6 (UBE2L6) and ubiquitin-conjugating enzyme E2 E1 (UBE2E1) were shown to be negative regulators of parkin recruitment (their knockdown increased parkin recruitment in the screen). Co-transfection of siRNAs targeting UBE2N and UBE2J2 mediated a statistically significant impairment in CCCP-induced parkin recruitment compared with the controls (siUBE2N + siUBE2J2 *versus* NTC, *p* < 0.0001; Fig. 3 (*A* and *B*)). The knockdown of UBE2N or UBE2J2 individually did not result in a statistically significant impairment in parkin recruitment as measured by the percentage of cells containing more than three parkin puncta. The screen hits were identified using the combined PCA and PLS analysis, a sensitive multiparametric method able to detect significant effects with low -fold change, and this may explain the inability to detect significance in this experiment. Targeting UBE2L6 and UBE2E1 alone or in combination did not result in significantly altered parkin recruitment when compared with the NTC. Subsequent interrogation of the primary and secondary screen data showed that there was a high degree of variation in the phenotypic measures (*e.g.* the percentage of cells containing more than three parkin puncta) (Fig. S6). This variation was particularly pronounced for UBE2E1 and therefore explains the apparent discrepancy in the data between initial screens and validation shown in Fig. 3*B*. siRNA-mediated knockdown of the UBE2s in the absence of CCCP had no effect on measures of parkin recruitment (data not shown).

To further validate and interrogate the effect of E2 ligase inhibition on parkin recruitment and mitophagy, we used Bay 11-7082 (27), a known pharmacological inhibitor of UBE2N. Pretreating cells with Bay 11-7082 prior to CCCP challenge (in the continued presence of Bay 11-7082) attenuated parkin recruitment in a dose-dependent manner (*F*_Bay(3,16)_ = 5.309, *p* < 0.01; Fig. 3 (*C* and *D*)). Combined siRNA-mediated knockdown of UBE2N and pharmacological inhibition with Bay 11-7082 showed no summative effect compared with Bay 11-7082 treatment alone, suggesting that the effect of Bay 11-7082 is mediated via inhibition of UBE2N and not one of its other targets (Fig. S7). Together, these data provide further evidence for the role of UBE2N in parkin-mediated mitophagy and demonstrate for the first time that pharmacological targeting of the ubiquitin pathway can be used to modulate mitophagy.

**Figure 4.**
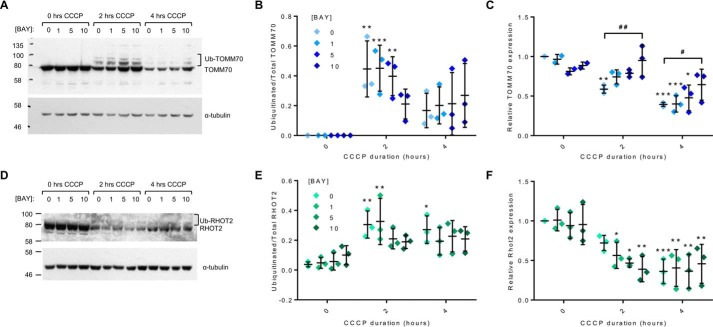
**Pharmacological inhibition of UBE2N modulates ubiquitination of OMM proteins following CCCP-induced PRKN recruitment.** *A–F*, H4-EGFP-PRKN cells were pretreated for 30 min with Bay 11-7082, and the ubiquitination and degradation of the OMM proteins TOMM70 and RHOT2 were assessed by Western blotting following CCCP treatment (10 μm). *A* and *D*, representative Western blots showing 70-kDa TOMM70 (*A*) and 80-kDa RHOT2 (*D*) and higher-molecular weight ubiquitinated species along with α-tubulin loading control. *B* and *E*, quantification of the ubiquitinated species expressed as a fraction of the total protein. *C* and *F*, quantification of total protein normalized to 0 μm Bay 11-7082 and 0 h CCCP. Bay 11-7082 reduces CCCP-mediated ubiquitination of TOMM70 and impairs its degradation. Bay 11-7082 also reduces CCCP-mediated ubiquitination of RHOT2, but the effect on its degradation is less clear. *n* = 3 for all experiments. *Lines*, mean ± S.D. (*error bars*). *, significant difference from 0-h CCCP at a given concentration of Bay 11-7082; #, significant difference between *highlighted pair*; */#, *p* < 0.05; **/##, *p* < 0.01; ***, *p* < 0.001.

**Figure 5.**
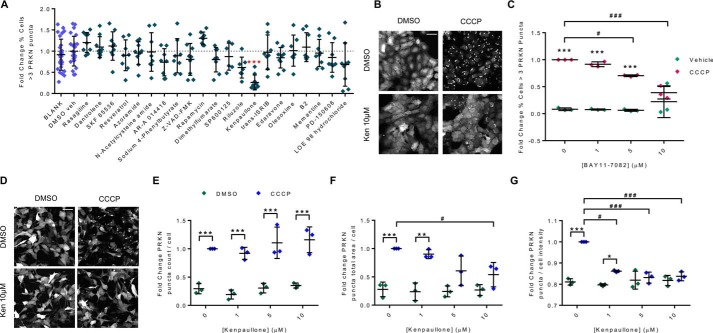
**Screening a neuroactive compound library identified kenpaullone as a modulator of PRKN recruitment.** *A*, H4-EGFP-PRKN cells were treated with the library compounds (10 μm) for 24 h and then treated with CCCP (10 μm, 2 h) to assay PRKN recruitment. Data show the percentage of cells with three or more PRKN puncta (from four independent plates). Analysis revealed a significant effect of compound (*F*_(22,224)_ = 3.468, *p* < 0.0001). Post hoc tests showed that kenpaullone significantly reduced the percentage of cells with PRKN puncta (*F*_(2,55)_ = 17.96, *p* < 0.001). *B* and *C*, H4-EGFP-PRKN cells were pretreated with kenpaullone for 24 h, and CCCP-induced PRKN recruitment was assayed as before. *B*, representative images of EGFP-PRKN. *C*, analysis of three independent plates revealed significant effects of kenpaullone and CCCP (*F*_ken(3,25)_ = 4.644, *p* < 0.05; *F*_CCCP(1,25)_ = 89.52, *p* < 0.0001; *F*_interaction(3,25)_ = 4.708, *p* < 0.01). *D–G*, the effect of kenpaullone on PRKN recruitment was also investigated in SH-SY5Y-EGFP-PRKN cells. *D*, representative images showing EGFP-PRKN in cells treated with kenpaullone and CCCP as indicated. *E*, treatment with kenpaullone did not change the mean number of parkin puncta per cell (*F*_ken(3,168)_ = 2.148, *p* = 0.1342). Total area of parkin puncta per cell (*F*_ken(3,16)_ = 3.451, *p* < 0.05) (*F*) and intensity of parkin puncta (normalized to the background fluorescence in each cell) (*F*_ken(3,16)_ = 19.55, *p* < 0.0001) (G) are both significantly reduced in cells treated with 1–10 μm kenpaullone compared with vehicle control (*n* = 3 independent plates). *E–G*, all three measures of PRKN recruitment showed a significant effect of CCCP. *Scale bar*, 50 μm. *Lines*, mean ± S.D. (*error bars*). *, significant difference between vehicle and CCCP; #, significant effect of kenpaullone between *highlighted pair*; *, *p* < 0.05; **/##, *p* < 0.01; ***/###, *p* < 0.001.

Ubiquitination and proteasomal degradation of OMM proteins by parkin is a key step in the degradation of mitochondria by mitophagy. This ubiquitination can be visualized by the appearance of higher-molecular-weight protein bands on a Western blot (Fig. 4, *A* and *D*) (28). We identified changes in the ubiquitination and expression levels of the OMM proteins TOMM70 and Ras homolog family member T2 (RHOT2/Miro2) when cells were pretreated with Bay 11-7082 prior to mitochondrial uncoupling with CCCP (Fig. 4). There was an increase in the ratio of ubiquitinated to total TOMM70 (Fig. 4, *A* and *B*) and RHOT2 (Fig. 4, *D* and *E*) following 2-h CCCP treatment that was attenuated in the presence of Bay 11-7082; similarly, there was a decrease in total protein expression of TOMM70 in the presence of CCCP that again was attenuated in the presence of Bay 11-7082, suggesting an impaired degradation of the protein (Fig. 4*C*). Analysis of the total levels of RHOT2 was less clear; there was a significant decrease in total protein levels following CCCP treatment but no effect of Bay 11-7082 (Fig. 4, *D* and *F*). In a second approach to identifying novel modulators of mitophagy and given the success of modulating this process with the compound Bay 11-7082, we conducted a screen of a targeted compound library. We selected pharmacological compounds known to modulate the genes/cellular pathways identified in our siRNA screen (oxidative stress, proteasome) and neuroactive compounds that acted via mechanisms relevant to neurodegeneration (ER stress, excitotoxicity, Nrf2 activation, and ion channels) (File S5). For each of the compounds chosen, there is strong evidence that they can modulate the relevant pathways both *in vitro* and *in vivo*, and several of the compounds are in clinical trials or in the clinic (*e.g.* riluzole). H4-EGFP-PRKN cells were treated with 10 μm concentrations of the neuroactive compounds for 24 h prior to the mitophagy assay (10 μm CCCP, 2 h in the presence of compound). Four compounds (GSK2606414, BIX, cu-ASTM, and TRC 051384) reduced the cell number by more than 20% and were therefore deemed toxic and excluded from further analysis (Fig. S8*A*). The effects on parkin recruitment were quantified, as before, revealing a significant effect of compound (*F*_(22,224)_ = 3.468, *p* < 0.0001) (Fig. 5*A*). Post hoc tests showed that kenpaullone, a cyclin-dependent kinase (CDK) and glycogen synthase kinase 3 (GSK3) inhibitor, significantly reduced the percentage of cells with more than three PRKN puncta (*p* < 0.001).

To validate this result, H4-EGFP-PRKN cells were treated with increasing doses of kenpaullone and assayed for PRKN recruitment (Fig. 5, *B* and *C*). Again, we observed a significant impairment of parkin recruitment in the presence of kenpaullone (*F*_ken(2,55)_ = 17.96, *p* < 0.0001) (Fig. 5*C*) and no toxicity as measured by cell count (Fig. S8*B*). The ability of kenpaullone to modify parkin recruitment to mitochondria was also investigated in EGFP-PRKN–expressing SH-SY5Y cells, a human neuroblastoma cell line with a dopaminergic phenotype commonly used as a neuronal model. SH-SY5Y-EGFP-PRKN cells showed CCCP-dependent PRKN recruitment to mitochondria (Fig. 5*D*). We noted that the recruitment was slower than seen in H4-EGFP-PRKN cells and that due to the tendency for individual cells to form one or two large puncta, measures of the total area or intensity of parkin puncta/cell gave a good readout, whereas measures based on the number of parkin puncta/cell were not optimal for detecting parkin recruitment (Fig. 5, *E–G*). Consistent with the results in H4-EGFP-PRKN cells, treatment of SH-SY5Y-EGFP-PRKN cells with kenpaullone attenuated parkin recruitment to the mitochondria following CCCP treatment as measured by the total area of parkin puncta/cell (*F*_(3,8)_ = 96.76, *p* < 0.0001; Fig. 5*F*) and the intensity of EGFP-PRKN within puncta compared with the cytoplasm (*F*_(3,8)_ = 67.17, *p* < 0.0001; Fig. 5*G*) without causing any cell toxicity (Fig. S8*C*). In the absence of CCCP, kenpaullone had no effect on PRKN puncta (Fig. 5, *D–G*).

**Figure 6.**
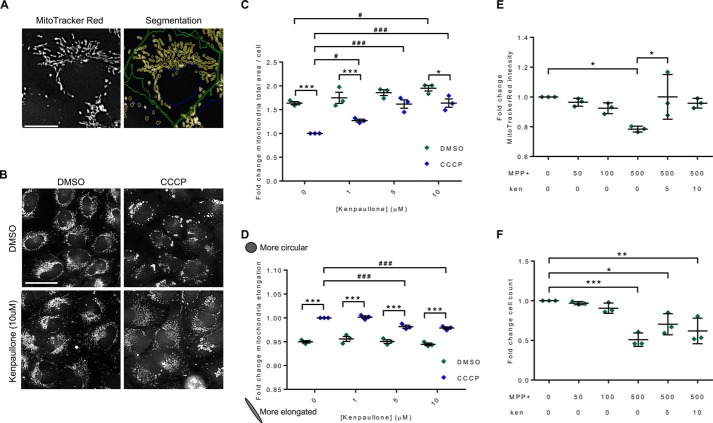
**Analysis of mitochondria in H4-EGFP-PRKN cells following treatment with CCCP, kenpaullone, and MPP+.** *A*, mitochondria stained with MitoTracker Red CMX Ros imaged using the Cy3 channel on the INCell Analyzer 2200 (×40 objective). Segmentation overlay using the INCell Work station software delineates nuclei (*blue*), cells (*green*), and mitochondria (*yellow*). *Scale bar*, 20 μm. *B*, representative images of mitochondria in H4-EGFP-PRKN cells treated with DMSO or kenpaullone (10 μm) for 24 h, followed by 2-h CCCP (10 μm) or vehicle control. *Scale bar*, 50 μm. *C*, quantification of the total area of the mitochondrial network/cell (*n* = 3) showed a significant effect of both CCCP and kenpaullone (*F*_CCCP(1,16)_ = 73.22, *p* < 0.0001; *F*_ken(3,16)_ = 20.14, *p* < 0.0001). *D*, quantification of the elongation of segmented mitochondria also showed a significant effect of both CCCP and kenpaullone. Note that greater values represent more rounded objects (*F*_CCCP(1,16)_ = 335.5, *p* < 0.0001; *F*_ken(3,16)_ = 12.79, *p* < 0.001). *E*, treatment with 500 μm MPP+ for 24 h resulted in significant reduction in cytoplasmic MitoTracker Red staining intensity compared with control. There was a significant increase in cytoplasmic MitoTracker Red staining intensity in MPP+-treated cells in the presence of kenpaullone at 5 μm compared with DMSO vehicle control (0 μm kenpaullone) (*n* = 3). *F*, cell count significantly decreased in the presence of 500 μm MPP+ compared with control. The addition of kenpaullone did not affect cell count in wells treated with 500 μm MPP+. *Lines*, mean ± S.D. (*error bars*). *, significant difference between DMSO and CCCP; #, significant difference between *highlighted pair*. */#, *p* < 0.05; **/##, *p* < 0.01; ***/###, *p* < 0.001.

To further investigate the effect of kenpaullone on mitochondria, we captured high-magnification (×40) images of H4-EGFP-PRKN cells using an InCell Analyzer 2200 and segmented the mitochondrial network using the organelle function (Fig. 6*A*). We were able to quantify CCCP-induced mitochondrial perinuclear clustering and fragmentation using the InCell Work station measures “organelle - total area” and “organelle - elongation” (Fig. 6, *C* and *D*). We also confirmed that there was no cell death (Fig. S9*A*) and that parkin puncta formed as expected (Fig. S9*B*). In the absence of CCCP, we observed that kenpaullone increased the total area of the mitochondrial network (*F*_CCCP(1,16)_ = 73.22, *p* < 0.0001; Fig. 6 (*B* and *C*)). Pretreatment of kenpaullone prior to CCCP treatment attenuated the CCCP-induced perinuclear clustering of mitochondria as measured by total area (*F*_ken(3,16)_ = 20.14, *p* < 0.0001; Fig. 6 (*B* and *C*)). Additionally, treatment with CCCP led to fragmentation of the mitochondrial network, quantified by an increase in the “elongation” measure (*F*_CCCP(1,16)_ = 335.5, *p* < 0.0001; Fig. 6*D*). Due to the nature of this measure, an increased value corresponds to more circular objects, and therefore the data show that CCCP caused the mitochondria to become more fragmented. Again, pretreatment of kenpaullone attenuated the effect of CCCP on mitochondrial fragmentation (*F*_ken(3,16)_ = 12.79, *p* < 0.001; Fig. 6*D*). We noted that the measures of the mitochondrial area in the 5 and 10 μm kenpaullone + CCCP cells were comparable with those in cells in the absence of both compounds, suggesting that kenpaullone could inhibit the effect of CCCP. To extend our findings, we differentiated human fetal neuronal precursor cells (see supporting Methods) to produce a heterogeneous population of neurons and glia (Fig. S9*C*). When these differentiated cells were treated with kenpaullone (5 μm for 24 h) prior to labeling with MitoTracker Red, we observed a dramatic increase in mitochondrial staining (Fig. S9*C*), a result that is consistent with the results observed in H4-EGFP-PRKN cells.

To further investigate the ability of kenpaullone to protect mitochondria, we treated H4-EGFP-PRKN cells with the complex I inhibitor MPP+. In the absence of kenpaullone, 24-h treatment with 500 μm MPP+ reduced the uptake of MitoTracker Red (*p* < 0.05), indicating a reduction in Ψ_m_, and this was accompanied by a reduction in cell number (*p* < 0.001) (Fig. 6, *E* and *F*). Co-treatment with kenpaullone (5 μm) rescued MitoTracker Red uptake (*p* < 0.05), indicating that these cells were able to maintain Ψ_m_, although there was no rescue of cell death.

**Figure 7.**
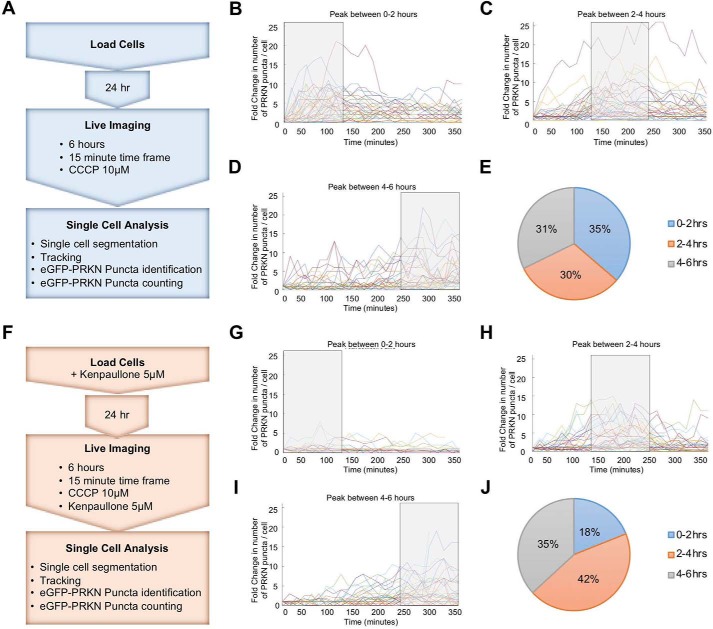
**EGFP-PRKN puncta quantification under CCCP and/or kenpaullone regime.** *A* and *F*, experimental scheme. H4-EGFP-PRKN cells were chip-loaded 24 h prior to time-lapse start and cultured in a tissue culture incubator with constant perfusion with control (*A*) or kenpaullone-containing (*F*) medium. The following day, the chip was placed on a widefield microscope, and cells were imaged every 15 min for 6 h, while constantly perfused with CCCP–conditioned (*A*) or CCCP + kenpaullone–conditioned (*F*) medium. *B–D* and *G–I*, change in the number of PRKN puncta/cell over time (each trace represents an individual cell). Cells were grouped according to the time of maximum EGFP-PRKN puncta formation, as represented by the *shaded boxes. B* and *G*, cells with maximum PRKN puncta between 0 and 2 h; *C* and *H*, cells with maximum PRKN puncta between 2 and 4 h; *D* and *I*, cells with maximum PRKN puncta between 4 and 6 h; *E* and *J*, pie charts showing the distribution of the cells according to their EGFP-PRKN puncta peak. *Lines* in *B–D* and *G–I*, H4-EGFP-PRKN puncta quantification of single cells.

Our results suggest that kenpaullone inhibited or possibly delayed the recruitment of parkin to mitochondria. To investigate this, we used a microfluidics platform that allows for the dynamic modulation of drug exposure with continuous monitoring of cell behavior (29–31). We made single-cell measures of parkin aggregation dynamics every 15 min for 6 h in H4-EGFP-PRKN cells (Fig. S10*A*), which were continuously perfused with medium containing CCCP (10 μm) (Fig. 7). Following time-lapse experiments, images were processed for single-cell EGFP-PRKN puncta quantification and cell tracking (Fig. S10*B*; see “Experimental procedures” for details about image processing and experimental set-up). H4-EGFP-PRKN cells continuously perfused with CCCP-conditioned medium were found to form EGFP-parkin puncta, as observed in our plate-based assays (Fig. S10, *B* and *C*). To determine the dynamics of the cellular response to CCCP exposure, we grouped cells (tracked individually) based on the time window (0–2, 2–4, 4–6 h) in which the maximum number of EGFP-parkin puncta was observed. Single cells equally distributed across three groups, with 35% of cells peaking between 0 and 2 h, 30% between 2 and 4 h, and 31% between 4 and 6 h (Fig. 7, *B–E*). We then tested the effect of kenpaullone on EGFP-parkin puncta formation. H4-EGFP-PRKN cells were perfused with kenpaullone (5 μm)-containing medium for 24 h before the start of the experiment. During the time-lapse experiments, cells were continuously perfused with medium containing both CCCP and kenpaullone (Fig. 7*F*). Tracking single cells and grouping them as above, we found that EGFP-parkin puncta formation occurred between 0 and 2 h in 18% of cells, whereas in the majority (42% of cells), the peak was between 2 and 4 h; the percentage of cells showing puncta between 4 and 6 h did not change as compared with CCCP treatment only (Fig. 7, *G–J*). Of note, in agreement with 96-well plate experiments (Fig. 5*C*), the number of EGFP-parkin puncta was reduced when kenpaullone was perfused in combination with CCCP (representative image in Fig. S10 (*C* and *D*)). Indeed, the maximum -fold increase in EGFP-parkin puncta in the presence of CCCP was 25 (Fig. 7, *B–D*), whereas, in the presence of kenpaullone, the maximum -fold change was 15 (Fig. 7, *G–I*). These results demonstrate the single-cell tracking and quantification of EGFP-parkin puncta and the controlled delivery of drugs in living cells and show that kenpaullone inhibits and delays parkin puncta formation.

## Discussion

We conducted a rigorous siRNA and drug screen to identify (i) novel genes and pathways important in regulating parkin recruitment and (ii) small neuroactive compounds and potential targets for therapeutic intervention in PD. We chose a library composed of siRNAs targeting G protein–coupled receptors, protein kinases, ion channels, phosphatases, proteases, ubiquitin-conjugating enzymes, and genes involved with apoptosis, senescence, nucleic acid binding, autophagy, DNA repair, and characterized nuclear receptors.

We used a bioinformatic approach to explore the hit genes and identified an overrepresentation of genes in the ubiquitin proteasome pathway by PANTHER pathway analysis (*proteasome 26S subunit, ATPase 5* (*PSMC5*); *proteasome 26S subunit, ATPase 6* (*PSMC6*); *UBE2L6*; *UBE2E1*; and *UBE2N*). This cluster was also revealed by STRING database analysis, which highlighted the interaction between the UBE2s UBE2E1, UBE2J2, UBE2L6, and UBE2N; the UBE3s F-box and WD repeat domain containing 12 (FBXW12) and ankyrin repeat and SOCS box–containing 9 (ASB9); and the proteasome genes *PSMC5*, *PSMC6*, and proteasome maturation protein (POMP). Ubiquitination of parkin (itself a UBE3) and OMM proteins, including mitofusin 1 (MFN1), mitofusin 2 (MFN2), Ras homolog family member T1 (RHOT1/Miro1), TOMM70, and voltage-dependent anion channel 1 (VDAC1), are known to be key steps in the mitophagy pathway (32, 33). Therefore, the identification of several UBE2s and UBE3s along with proteasomal subunits highlights the importance of this pathway in mediating parkin recruitment. UBE3s mediate the substrate specificity of the ubiquitination reaction, determining not only the substrate protein but also the type of ubiquitin linkage. UBE2s are responsible for charging the UBE3 with ubiquitin monomers and show specificity for specific UBE3s. UBE2s also confer specificity to the type of linkage.

Of the four UBE2s identified here, two (UBE2N and UBE2J2) showed a positive modulatory effect on parkin recruitment, and two (UBE2L6 and UBE2E1) had a negative effect. This is in agreement with, and adds to, the results of a previous study, which demonstrated a positive role for UBE2N, ubiquitin-conjugating enzyme E2 L3 (UBE2L3), and ubiquitin-conjugating enzyme E2 D2/3 (UBE2D2/3) in parkin translocation, ubiquitination, and degradation of OMM proteins and recruitment of p62 to mitochondria and a negative role for ubiquitin-conjugating enzyme E2 R1 (UBE2R1) (28). A plausible interpretation of these results is that UBE2s act as positive modulators to charge parkin with Ub and/or facilitate the ubiquitination of substrates, including parkin itself and OMM proteins. Existing evidence suggests that UBE2N is primarily responsible for the building of Lys-63–linked Ub chains (a linkage that is a well-established method for directing cargo toward autophagosome-mediated degradation rather than proteasomal) but is less important for the initial charging of parkin with Ub (28, 34). UBE2s that negatively regulate mitophagy may play a role in the proteasomal degradation of cleaved PINK1; in this scenario, their knockdown would promote PINK1 accumulation at the mitochondria (35). In addition, there are additional mitochondrial resident UBE3s, such as mitochondrial E3 ubiquitin protein ligase 1 (MUL1), that play a role in mitophagy (36) and whose activity would also be dependent on UBE2s and may be modulated by the activity of recruited PINK1/parkin.

We observed a cooperative effect of the positive modulators UBE2N and UBE2J2 on the recruitment of parkin to mitochondria. This is in agreement with other studies that have shown greater deficits when two or more UBE2s are knocked down compared with knockdown of individual genes (34).

In addition to the UBE2 family members, two E3 ubiquitin ligases (ASB9 and FBXW12) were included on our hit list. ASB9 has been shown to ubiquitinate mitochondrial creatine kinase (CKMT1) and thus target it for degradation (37, 38). CKMT1 is a gatekeeper of the mitochondrial permeability transition pore, and loss of CKMT1 results in depolarization of mitochondria and induction of apoptosis. The second UBE3 subunit we identified was FBXW12; this protein has not been well-characterized, but recently the rs9614 C variant has been associated with a reduced risk of PD (39). Interestingly, alterations in both ASB9 and FBXW12 have also been associated with Alzheimer's disease (40–42). Current models of parkin recruitment suggest the accumulation of PINK1 (on depolarized mitochondria) mediates the phosphorylation of ubiquitin and parkin (19). This basal activation then stimulates a feed-forward loop resulting in further conjugation of ubiquitin to mitochondria and PRKN recruitment. Hence, it is possible that ASB9 and FBXW12 play a role in mediating the PINK1-mediated conjugation of ubiquitin to mitochondria and the subsequent amplification of parkin recruitment.

Ubiquitination of proteins can target them for degradation by the proteasome, and we identified two protease subunits (PSMC5 and PSMC6) among our hit genes along with POMP. Both PSMC5 and PSMC6 have been shown to interact with parkin following CCCP-induced mitochondrial depolarization. Furthermore, PSMC5 displayed parkin-dependent ubiquitination following CCCP treatment (33). In addition, PSMC6 has been shown to interact with PINK1 (43). We also demonstrated that inhibiting UBE2N with a pharmacological compound (Bay 11-7082) replicated the effects of the siRNA-mediated knockdown both on PRKN recruitment and ubiquitination and degradation of OMM proteins, further supporting a role for UBE2s in mitophagy (*i.e.* subsequent to PINK1-parkin recruitment) via the ubiquitination and degradation of OMM proteins.

Our analyses also identified a number of genes involved in regulating cellular oxidative state as being able to modulate PRKN recruitment. GSH reductase (GSR), a positive modulator of parkin recruitment, was identified as a node gene by our STRING database analysis. GSR catalyzes the reduction of GSSG to GSH and so acts as an important antioxidant. Interestingly, GSH peroxidase 6 (GPX6), which catalyzes the reverse reaction, mainly oxidation of GSH to GSSG, was identified as a negative modulator. Together, these hits strongly support a positive role for GSH in parkin recruitment. Reduced GSH levels in the substantia nigra have long been associated with PD (44, 45), and although not thought to be a primary cause of the disease, the decrease in GSH is hypothesized to exacerbate neuronal damage, particularly by increasing oxidative stress (44). GSH is one of most abundant cellular antioxidants. When GSH levels are decreased, the cell struggles to remove reactive oxygen species, resulting in increased reactive oxygen species levels that lead to DNA damage, lipid peroxidation, and mitochondrial damage. Our analyses also found that genes involved in regulating copper (*SLC31A1, CP,* and *AOC1*) and zinc metabolism (*SLC30A4*) regulated parkin recruitment (46). These essential metals act as enzyme co-factors, and their dysregulation has been shown to increase oxidative stress and activate the SP1/AP1 and TGF-β transcriptional pathways (46). Our screen also identified transcription factors (SP1 and SNW1) involved in regulating the SP1 and TGF-β pathways. In addition, a number of genes known to regulate glucose metabolism (*HK3, HK2,* and *PPARG*) were also found in our screen. Together, these findings suggest that specific enzymatic, glucose, antioxidant, and transcriptional systems impact the regulatory systems controlling mitophagy, a simple explanation being that mitochondria when not coping with additional stress (*e.g.* oxidative) have more patent quality control systems.

In addition to the large druggable genome siRNA screen, we conducted a screen of a small selected compound library. This screen identified the CDK (specifically, CDK1, CDK2, and CDK5) and glycogen synthase kinase (GSK3) inhibitor kenpaullone as a negative modulator of parkin recruitment and downstream mitophagy processes. Investigation of the mechanisms underlying the inhibition of parkin revealed that in the absence of CCCP, treating H4-EGFP-PRKN cells with kenpaullone enlarged the area of the mitochondrial network. Furthermore, kenpaullone rescued the CCCP-induced perinuclear clustering and fragmentation of mitochondria, therefore maintaining a healthy network throughout the cytoplasm. Further preliminary experiments showed that treatment with kenpaullone dramatically increased the mitochondrial network in human primary neurons and glia derived from fetal neuroprogenitor cells. Kenpaullone may therefore be promoting healthy mitochondrial dynamics and making them more resistant to pathogenic change and/or stimulating mitochondrial biogenesis while repressing parkin recruitment. That the CDK-inhibitory actions of kenpaullone may represent a therapeutic avenue for PD is supported by recent data. CDK5 activation has been implicated in PD; in particular, it has been found within Lewy bodies and has been shown to mediate DA neurodegeneration in mouse models of PD (47, 48). In the developing nervous system, activation of CDK5 by p35 is important for neurite outgrowth and neuronal migration. However, cleavage of p35 by calpains to p25 leads to aberrant activation of CDK5 and has been associated with neurodegeneration in PD and Alzheimer's and Huntington's diseases (49). More specifically with regard to PD, p25/CDK5 has been demonstrated to regulate the ubiquitin ligase activity of parkin via phosphorylation of serine 131 (50). Additionally, CDK5 can regulate stress-induced autophagy via phosphorylation of SH3 domain–containing GRB2-like, endophilin B1 (SH3GLB1/EndoB1/Bif1), which in turn recruits the UVRAG/Beclin1 complex (51). CDK1 and CDK5 also phosphorylate the PD-associated protein, vacuolar protein-sorting 34 (VPS34), inhibiting its interaction with Beclin1 and thus attenuating autophagy (52). Another major target of kenpaullone, GSK3β, has also been implicated in PD (53, 54), with polymorphisms in the GSK3β gene being associated with PD risk in the East Asian population (55). Likewise, mitochondrial GSK3β exacerbates the effects of rotenone and MPP+ and increases oxidative stress (54). Both CDK5 and GSK3β are negative regulators of the neuroprotective transcription factor myocyte enhancer factor 2 (MEF2) (56). In addition to promoting neuronal survival via transcription of nuclear genes, MEF2 can localize to the mitochondria and regulate transcription of mitochondrial DNA–encoded genes as well as complex I activity (57). Furthermore, MEF2 has been shown to be reduced in mouse models of PD and post-mortem PD patient brain samples (57). She *et al.* (57) proposed that the loss of mitochondrial MEF2 can sensitize neurons to toxic/stress insults and thus play a role in the development of PD. Together, these reports suggest synergistic mechanisms by which the inhibition of CDKs and/or GSK3 by kenpaullone may alter parkin recruitment. Overall, these findings suggest specific pathways and pharmacological compounds to be targeted and developed for the treatment of PD.

## Experimental procedures

### Cell culture and generation of stable cell lines

H4 cells were used as they are of human origin and are derived from a central nervous system tissue (neuroglioma); additionally, they can be readily cultured, have high transfection efficiency, and are suitable for high-content imaging. H4 cells were maintained in Dulbecco's modified Eagle's medium (Sigma–Aldrich, D6546) supplemented with 10% fetal bovine serum (Life Technologies, Inc., 10500064), 2 mm l-glutamine (Sigma–Aldrich, G7513), and 5000 units/ml penicillin/10 μg/ml streptomycin (Sigma–Aldrich, P4458). SH-SY5Y cells were maintained in Dulbecco's modified Eagle's medium/F-12 (1:1) (Thermo Fisher Scientific, 21331020) supplemented with 10% fetal bovine serum (Life Technologies, 10500064), 2 mm l-glutamine (Sigma–Aldrich, G7513), and 5000 units/ml penicillin/10 μg/ml streptomycin (Sigma–Aldrich, P4458). All cells were maintained in a humidified incubator at 37 °C, 5% CO_2_.

H4 and SH-SY5Y cells stably expressing EGFP-PRKN were created using a lentiviral vector. EGFP-PRKN (a kind gift from Dr. Jon Lane, University of Bristol) was cloned into a third-generation lentiviral backbone, and viral particles were produced by co-transfection of HEK293T cells as described previously (58). Cells were transduced with the virus and, after repeated passaging, sorted by FACS to produce a cell line expressing a consistent level of EGFP-PRKN.

### EGFP-parkin mitochondrial recruitment assay for siRNA screening

For the primary screen, the OnTarget Plus druggable genome Smartpool siRNA (4 siRNAs/gene) library targeting 7500 genes (Thermo Fisher Scientific, G-104605) was used for the primary screen. H4-EGFP-PRKN cells were seeded in black-walled 96-well plates with optical bottoms (Corning, 3904) at a density of 4000 cells/well and 24 h later transfected with 6 pmol of siRNA/well using Lipofectamine2000 (Thermo Fisher Scientific, 11668019). An NTC smart pool (Thermo Fisher Scientific, D-001810-10-50) was used to control for transfection (wells A1, B1, C1, D1, E12, F12, G12, and H12), and a PINK1-targeting smart pool was used as a positive control (wells A12, B12, C12, D12, E1, F1, G1, and H1) (Thermo Fisher Scientific, L-004030-00-0050). 48 h post-transfection, MitoTracker Red CMXRos (100 nm; Thermo Fisher Scientific, M7512) was added to label mitochondria. Mitophagy was induced by adding 10 μm CCCP (Sigma–Aldrich, C2759) for 2 h. Cells were fixed with 4% paraformaldehyde, and the nuclei were labeled with Hoechst (1 μg/ml). Transfections and assays were performed in triplicate.

For the secondary screen, three individual Silencer Select siRNAs (Ambion, Thermo Fisher Scientific) were used per gene. An improved “controls” plate layout (59) involved the repositioning of the NTC siRNA oligonucleotide (wells A2, A12, C5, C8, D10, E3, F5, F8, H2, and H12) and PINK1-targeting siRNA oligonucleotide (wells A1, A11, C4, C9, F4, F9, H1, and H11). 24 h after seeding, H4-EGFP-PRKN cells were transfected with 0.25 pmol/siRNA well using Lipofectamine2000. The assay was performed as for the primary screen except that six replicate transfections and assays were performed for each plate.

### High-content imaging

Images were captured using an InCell Analyzer 1000 (GE Healthcare). A ×10 objective was used, and four fields of view were captured per well. The InCell Investigator software was used to segment and quantify the images as follows. A top hat algorithm was used to identify cell nuclei (Hoechst stain). Cell boundaries were defined using a multiscale top hat algorithm analysis on the MitoTracker Red CMXRos signal. Parkin puncta were identified using the organelle feature with a multiscale top hat algorithm analysis of the EGFP signal. The output of the InCell Investigator software for each plate consisted of a set of tiff files of the images of the microscope taken at different fields and a xls file with the associated numerical data. The xls file included the data of the individual fields and also the summary data of all of the fields and wells in different sheets. The numerical data were recorded using 84 different parameters as described in File S6. The initial primary screen data set consisted of three replicates of 98 different plates. An additional 78 plates were analyzed after the quality control (QC) stage. The secondary screen data set consisted of six replicates of 12 plates.

### Data analysis workflows

The data were processed and analyzed using the open source software R (60). For the data analysis, we used the well summary data provided by the InCell Investigator software and three different workflows that take into account different parameter selection approaches to compute a hit score for each gene: univariate workflow, PCA workflow, and PLS workflow. These three workflows can be summarized as having four parts each: row/column artifact correction, quality control of the plates, parameter selection, and hit selection. The only difference in these workflows is in the parameter selection stage.

### Row/column artifact correction

The preprocessing stage included a step aimed at reducing the potential spatial artifacts that can arise in siRNA screenings due to the geometry of the plates (59, 61). We applied the method suggested by Zhang (59) to identify and correct these systematic spatial artifacts. This method is based on a fitting robust linear model for each plate, where the variable to be corrected is fitted against the row and column numbers. If a systematic effect exists in the plate, it will be captured in the slope of such a model. The systematic artifacts are corrected by removing the slope of the robust linear model from the data.

### Quality control of the plates

To evaluate the quality of the plates and remove the ones that contain unreliable data, we computed a robust plate-wise QC score given by the following,

(Eq. 1) $$SSMD_{QC}=\frac{median\left(Control_{+}\right)-median\left(Control_{-}\right)}{\sqrt{{\left(MAD\left(Control_{+}\right)\right)}^{2}+{\left(Mad\left(Control_{-}\right)\right)}^{2}}}$$

where MAD is the median absolute deviation, and it is defined as follows.

(Eq. 2) $$MAD=1.4826\cdot median\left(\left|x_{ij}-median\left(x\right)\right|\right)$$

The indices *i* and *j* refer to the row and column of the well, whereas the constant 1.4826 makes the MAD comparable with the S.D. (62, 63). The SSMD_QC_ cut-off value was set to −2, as suggested for very strong positive controls (59). Therefore, all of the plates with an SSMD_QC_ greater than −2 in the univariate workflow were tagged as being of poor quality and were removed from the data set.

### Hit selection

We evaluated the effect of silencing a certain gene *i* for a given variable by using a SSMD score per gene, which gives an estimate of the effect of silencing a gene, compared with the negative control for the same plates using the following,

(Eq. 3) $$SSMD_{i}=\frac{median\left(well_{i}\right)-median\left(Control_{-}\right)}{\sqrt{\frac{1}{2}{s^{2}}_{well_{i}}+\frac{1}{2}\bar{{s_{0}}^{2}}}}$$

where *s*_0_^2^ is the median of the squares of the S.D. values for all the plate replicates, *s*_well *i*_^2^ is the square of the S.D. of the well *i*, and *well_i_* is the readout of the *i*th well that we want to use to evaluate whether the silenced gene in that well gives an output different from the negative control wells (*Control*_−_).

### Description of the workflows

Three different strategies were followed to select the relevant parameters that led to three different workflows for the data analysis.

#### 

##### Univariate workflow

After row/column artifact compensation and the QC step, three parameters were selected as parkin translocation indicators (Fig. S11). We performed this selection according to previous literature, where it was shown that these parameters are good readouts for PRKN translocation in siRNA high-content screenings (20). These parameters were: percentage of cells with more than three parkin puncta, area of parkin per cell, and parkin puncta/cell intensity. Each parameter was fed to the robust SSMD formula, and a hit list for each parameter was obtained. We defined a threshold of SSMD > 3 or SSMD < −3 to identify the hits.

##### PCA workflow

The row/column artifact compensation was performed at the beginning of the workflow. We selected a subset of six potential parameters that could be used as readouts for parkin translocation. Through the plotting of the positive and negative control data sets for these six parameters (Fig. S12), we identified three that show a different pattern for positive and negative controls and, therefore, could be used to evaluate the level of parkin translocation. These parameters were percentage of cells with more than three parkin puncta, area of parkin puncta per cell, and parkin puncta/cell intensity. This was followed by QC and SSMD calculation for each of the three parameters as described above. In the last step of the pipeline, the three SSMD values per gene were combined using a PCA model to extract the main source of variance in the data and reduce the dimensionality to just one parameter per gene as follows.

*X* was defined as a matrix having as many rows as the number of genes screened and three columns (one for each of the selected parameters in this workflow). Application of a singular value decomposition generated the following,

(Eq. 4) $$X=UDV^{T}$$

where *U* and *V* were the left and right singular vectors, respectively, and *D* was a diagonal matrix with the corresponding singular values. Rearrangement of this equation resulted in the generation of a metric for PCA decomposition,

(Eq. 5) $$X=HW^{T}+E$$

where *W* was the loading matrix, *H* was the scores matrix, and *E* was the residual matrix. The dimensionality of the *H* and *W* matrices depends on the number of latent components that are assumed to be playing a role in the variance of the data. In our case, it was assumed that there was only one component capturing the differences between the positive and negative controls and, therefore, the matrix *H* only had one column and as many rows as genes. We used each of the scores in the vector *H* as a consensus SSMD value (PCAscore) that combined the three SSMD scores computed in the previous steps. A threshold was set at SSMD > 3 or SSMD < −3 to obtain the list of hits in the primary screen. The secondary screen consisted of three nonpooled different siRNAs targeting the candidate genes. This caused a drop in the effect sizes of the readouts that led us to set a significance threshold for the secondary screening of SSMD > 1.3 or SSMD < −1.3.

##### PLS workflow

The PLS workflow was the most parameter-agnostic of the workflows that we applied, as it lacked manual selection of parameters. The correlation structure of the initial screening data shows groups of variables that are highly correlated (Fig. S12). The first step of this workflow was the row/column compensation step. The next step in the workflow involved the detection of outliers, which was based on Hotelling's *T*^2^ for control values. To perform a first selection of relevant features, a *t* test for each parameter was performed using the positive and negative control data. At a false discovery rate cut-off of 0.05, we found that 65 of the 84 parameters were significant. These were used to build a PLS model using the control data.

Given a set of *M* variables measured for *N* samples expressed as a data matrix *X* and the readouts of *L* observations for the same *N* samples in another data matrix *Y*, PLS performed a decomposition of both matrices,

(Eq. 6) $$X=TP^{T}+E$$

(Eq. 7) $$Y=UQ^{T}+F$$

where *T* and *U* were the *X*-scores and *Y*-scores, respectively, *P* and *Q* were the *X*-loadings and *Y*-loadings, and *E* and *F* were the *X*-residuals and *Y*-residuals. The decomposition was computed so that the covariance between *T* and *U* was maximized. In this case, the matrix with the readouts of the 65 parameters for the control wells was matrix *X*, and matrix *Y* was the matrix containing the labels of the controls (NTC or PINK1). We fitted this model using cross-validation to avoid overfitting and used it to predict scores for the noncontrol wells. These scores were fed to the QC stage as described above to remove misleading plates. The data of the plates that passed were used to build another PLS model. The scores of this PLS model were used as *well_i_* in the hit selection step as described above. Finally, we set a threshold at SSMD > 3 or SSMD < −3 to obtain the list of hits in the primary screen. The secondary screening consisted of three nonpooled different siRNAs targeting the candidate genes. This caused a drop in the effect sizes of the readouts, resulting in the significance threshold for the secondary screening being set to SSMD > 1.3 or SSMD < −1.3.

### Gene expression in H4 cells

To extract the list of genes that were expressed in H4 cells, we queried the Gene Expression Omnibus (https://www.ncbi.nlm.nih.gov/geo/) for control H4 cells. Eight samples were identified that fulfilled these conditions, and these samples were downloaded. The GEO IDs of the samples were GSM203415, GSM203416, GSM203417, GSM549595, GSM887031, GSM1050692, GSM1050693, and GSM1050694. An extra three samples were downloaded from Array Express (https://www.ebi.ac.uk/arrayexpress/)^3^ (68). The sample IDs for these samples were H4_SS331525_HG-U133_Plus_2_HCHP-167398, H4_SS331526_HG-U133_Plus_2_HCHP-167399, and H4_SS331527_HG-U133_Plus_2_HCHP-167400. These files were analyzed using the expresso function of the affy package in R (64). We used RMA to correct the background and quantile normalization and median polish as a method to summarize the probes. The probability density of the data had a bimodal shape due to the mixture of expressed and nonexpressed genes. To deconvolute both distributions, we fitted a Gaussian mixture model made of two Gaussian distributions using expectation maximization and the R package mclust (65, 66).

The Gaussian with the lower mean value corresponded to the nonexpressed distribution, whereas the Gaussian distribution with the higher mean corresponded to the distribution of expressed genes. We sampled 10,000 points from the distribution of nonexpressed genes, and we computed gene-wise *t* tests between the expression of the genes and these sampled points. The *p* values of such tests were corrected by multiple testing. The genes whose corrected *p* values were under or equal to 0.05 were considered not to have been generated from the distribution of nonexpressed genes. As a gene is either expressed or nonexpressed (there are only two distributions originating the data, given that all of the samples are H4 controls), the significant genes of such tests were considered as expressed genes. The rest of the genes were considered nonexpressed in the H4 cell line.

### Parkin interactor enrichment

To evaluate whether the list of hits that were identified out of the primary screening were enriched for parkin interactors, a hypergeometric test using a list of genes previously reported to be interactors of parkin was performed (24).

### qPCR validation of siRNA knockdown

H4-EGFP-PRKN cells were seeded in 12-well plates at a density of 50,000 cells/well. Cells were transfected with 3 pmol of siRNA/well using Lipofectamine2000 (2 μl/well). 48 h post-transfection, RNA was extracted using the ReliaPrep^TM^ RNA Cell and Tissue Miniprep Systems (Promega, Z6212). 1 μg of RNA was reverse-transcribed to cDNA using the High-Capacity cDNA Reverse Transcription Kit (Thermo Fisher Scientific, 4368814). To assess the degree of mRNA knockdown, qPCR was performed using Power SYBR® Green PCR Master Mix (Thermo Fisher Scientific, 4367659) and specific primers (300 nm) (File S7). All transfections were performed in triplicate, and three technical replicates of each PCR were run. Data were analyzed using the ΔΔ*Ct* method with β-actin serving as the endogenous control and NTC transfection as the normalizer.

### Parkin recruitment assays

These individual assays were performed in an identical manner to those for the primary and secondary screens. For these siRNA experiments, H4-EGFP-PRKN or SH-SY5y-EGFP-PRKN cells were seeded in black-walled 96-well plates and transfected with siRNAs for 48 h. They were then labeled with MitoTracker Red CMXRos and treated with CCCP (10 μm) for 2 h, followed by fixation and Hoechst staining. For pharmacological compound experiments, cells were pretreated with the compound prior to CCCP treatment in the continued presence of the compound. Stock solutions of Bay 11-7082 (Sigma–Aldrich, B5556, 25 mm) and kenpaullone (Sigma–Aldrich, K3888, 5 mm) were prepared in DMSO, and serial dilutions were prepared in medium. Plates were imaged and quantified using the InCell Analyzer as described above.

### Western blotting

To assess the ubiquitination of OMM proteins, H4-EGFP-PRKN cells were seeded in 12-well plates at a density of 55,000 cells/well. 48 h post-transfection, mitochondrial recruitment of EGFP-parkin was induced by treatment with CCCP (10 μm). For pharmacological compound experiments, these cells were pretreated with the compound (see “Parkin recruitment assays”) prior to CCCP (10 μm) treatment in the continued presence of the indicated compound. Cells were lysed in radioimmune precipitation assay buffer, and the protein concentrations were determined using a BCA assay (Thermo Fisher Scientific). Typically, 20 μg of protein was mixed with SDS sample buffer, heat-denatured, and loaded onto 10% polyacrylamide gels followed by electrophoresis in a Tris-glycine buffer. Proteins were transferred to a polyvinylidene difluoride membrane, which was subsequently blocked with 5% milk powder in PBS and probed with primary antibodies against TOMM70 (Proteintech 14528-1-AP; 1:500 in 1% milk PBS), RHOT2 (Proteintech 11237–1-AP; 1:500 in 1% milk PBS), and α-tubulin (Sigma T5168; 1:2000 in 1% milk PBS). Blots were then probed with species-specific horseradish peroxidase–conjugated secondary antibodies (Amersham Biosciences, NA931/NA934) before development with enhanced chemiluminescence (TOMM70 and RHOT2: ECL Prime GE Healthcare, RPN2232; α-tubulin Thermo Scientific SuperSignal West Pico, 34580). ImageJ (National Institutes of Health) densitometry was used to measure semi-quantifiably the expression of each protein. Expression of native and ubiquitinated forms were normalized to that of α-tubulin, and the ratio of ubiquitinated to total protein was calculated.

### Compound library screen

A library of neuroactive compounds was selected and a master plate (96-well) was prepared containing a 5 mm compound of each compound (in DMSO). Compounds were stamped into daughter plates and diluted to 50 μm with culture medium. This was then further diluted 1:5 by the addition of 25 μl to cells seeded in 100 μl of medium 96-well plates, giving a final concentration of 10 μm and final DMSO concentration of 0.2%. Cells were incubated with the compounds for 24 h prior to CCCP treatment (10 μm, 2 h) in the continued presence of 10 μm compound. Cells were fixed and Hoechst-stained, and plates were imaged and quantified using the InCell Analyzer as described above. Compounds that reduced cell number by >20% were excluded from analysis of parkin puncta.

### Mitochondrial network analysis

To image and analyze the mitochondrial network, H4-EGFP-PRKN cells were seeded in black-walled 96-well plates with optical bottoms (Greiner, 655090). Cells were treated with kenpaullone or vehicle control for 24 h prior to loading with MitoTracker Red CMXRos and treatment with CCCP. Images were captured on an InCell Analyzer 2200 using the ×40 objective and two-dimensional deconvolution mode. The InCell Investigator software was used to segment and quantify the images as follows. A top hat algorithm was used to identify cell nuclei (Hoechst stain). Cell boundaries were defined using a multiscale top hat algorithm analysis on the MitoTracker Red CMXRos signal. Mitochondria were identified using the organelle feature with a multiscale top hat algorithm analysis of the MitoTracker Red CMXRos signal.

### Microfluidics/microscopy-based time lapses

Microfluidics time lapses were performed using the device designed in the laboratory of Prof. Jeff Hasty (University California San Diego, La Jolla, CA) (67) and optimized for mammalian cell growth, ensuring controlled flow perfusion, CO_2_ diffusion, and minimal stress. Pelleted cells from a subconfluent 75-cm flask were resuspended in 500 μl of complete medium and chip-loaded as in Ref. 30. Cells were cultured for 24 h in a tissue culture incubator (5% CO_2_, 37 °C) under constant perfusion with standard or kenpaullone-containing medium. Medium was perfused with a syringe directly connected to port 2 via 24-gauge PTFE tubing (Cole-Parmer Inc.). Port 5 was used for waste medium, whereas ports 1, 6, and 7 were plugged to avoid medium spillage. The day after, the device was perfused with medium containing the nuclear dye Hoechst (1 μm) for 30 min before being transferred onto the widefield microscope for the time lapse. The actuation system consists of two motor-controlled syringes (http://biodynamics.ucsd.edu/dialawave/)^3^ connected to ports 6 and 7. One syringe contained standard medium, and the second contained either CCCP– or CCCP + kenpaullone–supplemented medium. Ports 1, 2, and 5 are also connected to static syringes working as waste tanks. The correct flow of the medium was measured using a red dye (sulforhodamine 101 from Sigma) added to the CCCP/kenpaullone–containing syringe.

The microscopy platform consisted of a Leica DMi8 inverted microscope equipped with the digital camera Andor iXON 897 ultra back-illuminated EMCCD (512 × 512, 16-μm pixels, 16-bit, 56 fps at full frame) and an environmental control chamber (PeCon) for long-term temperature control and CO_2_ enrichment. The adaptive focus control ensures that the focus is maintained during the entire time-course experiment. The experimental set-up includes consecutive acquisition in three channels (phase-contrast and green and red fluorescence) with a ×40 objective every 15 min. A power lamp was set at 33% with an exposure time of 500 ms.

### Microfluidics/microscopy image segmentation

Single cells were segmented using an image-processing algorithm recognizing the nucleus, in the blue spectrum (Hoechst), as an ellipse by means of the MATLAB function regionprops (Mathworks Matlab R2018b).

For puncta identification, we first designed a threshold in the green spectrum (EGFP-parkin) to generate a binary image selecting only pixels belonging to EGFP-parkin puncta by using the MATLAB function imextendmax (Mathworks Matlab R2018b); then we selected a threshold on puncta area size (5 < area < 2000). Finally, identified EGFP-parkin puncta were attributed to the cell they belong to by measuring the relative distance of the EGFP-parkin puncta centroid to the nucleus center. For cell attribution, puncta centroid distance from the nucleus center has to be less than or equal to the nucleus diameter. A summary of the segmentation pipeline is shown in Fig. S8*B*.

### Statistical analysis

Experimental data were analyzed in GraphPad Prism by one-way or two-way analysis of variance as appropriate with post hoc tests. *F* values are reported as *F*_condition(_*_x,y_*_)_, where *x* represents the condition degrees of freedom and *y* is the error degrees of freedom.

## Author contributions

H. L. S., N. B., F. F.-A., E. P., L. P., G. S., N. A., L.-F. W., L. Magini, L. Marucci, G. A. O., S. C., J. P., P. M., and J. B. U. investigation; H. L. S., N. B., F. F.-A., L. Marucci, G. A. O., S. C., J. P., P. M., and J. B. U. methodology; H. L. S., F. F.-A., E. P., L. Marucci, G. A. O., S. C., J. P., P. M., and J. B. U. writing-original draft; F. F.-A. and E. P. formal analysis; N. A., L. Magini, L. Marucci, G. A. O., S. C., J. P., P. M., and J. B. U. funding acquisition; N. A. project administration; L. Magini, L. Marucci, G. A. O., S. C., J. P., P. M., and J. B. U. supervision; L. Marucci, G. A. O., S. C., J. P., P. M., and J. B. U. conceptualization.

## Supplementary Material

Supporting Information

## Footnotes

3 Please note that the JBC is not responsible for the long-term archiving and maintenance of this site or any other third party hosted site.

## Footnotes

2 The abbreviations used are:

PD: Parkinson's disease
GPX6: glutathione peroxidase 6
GSK3: glycogen synthase kinase 3
GSR: glutathione-disulfide reductase
HK2: hexokinase 2
MEF2: myocyte enhancer factor 2
MPP+: 1-methyl-4-phenylpyridinium
NTC: non-targeting control
OMM: outer mitochondrial membrane
PCA: principal component analysis
PINK1: PTEN-induced putative kinase 1
PLS: partial least squares
PRKN: parkin
PSMC6: proteasome 26S subunit
RHOT2: Ras homolog family member T2 (Miro2)
SSMD: strictly standardized mean difference
TOMM70: translocase of outer mitochondrial membrane 70
UBE2: E2 ubiquitin-conjugating enzyme
Ψ_m_: mitochondrial membrane potential
CCCP: carbonyl cyanide 3-chlorophenylhydrazone
EGFP: enhanced green fluorescent protein
qPCR: quantitative PCR
DA: dopaminergic
CDK: cyclin-dependent kinase
POMP: proteasome maturation protein
SH3: Src homology 3
QC: quality control.
