# Supplementary material for: A dual druggable genome-wide siRNA and compound library screening approach identifies modulators of parkin recruitment to mitochondria
Source: J Biol Chem. 2020 Jan 7;295(10):3285–300. doi: 10.1074/jbc.RA119.009699 (PMC7062187; doi:10.1074/jbc.RA119.009699)
Supplement: Supporting Information [file supp_295_10_3285__index.html]

A dual druggable genome-wide siRNA and compound library screening approach identifies modulators of parkin recruitment to mitochondria — Identifying novel gene and drug modulators of mitochondria — A dual druggable genome-wide siRNA and compound library screening approach identifies modulators of parkin recruitment to mitochondria — Identifying novel gene and drug modulators of mitochondria — Supporting Information 

# A dual druggable genome-wide siRNA and compound library screening approach identifies modulators of parkin recruitment to mitochondria

## Supporting Information

- Supporting Information (to be published online) - Supporting file1
- Supporting Information (to be published online) - supporting file 2
- Supporting Information (to be published online) - Supporting file 3
- Supporting Information (to be published online) - Supporting file 4
- Supporting Information (to be published online) - Supporting File 5
- Supporting Information (to be published online) - Supporting file 6
- Supporting Information (to be published online) - Supporting file 7
- Supplemental figures 1-10 - Supplemental figures
