# Supplementary material for: A dual druggable genome-wide siRNA and compound library screening approach identifies modulators of parkin recruitment to mitochondria
Source: J Biol Chem. 2020 Jan 7;295(10):3285–300. doi: 10.1074/jbc.RA119.009699 (PMC7062187; doi:10.1074/jbc.RA119.009699)
Supplement: Supporting Information [file supp_RA119.009699_153385_3_supp_447762_q2tg8c.pptx]

## Slide 1
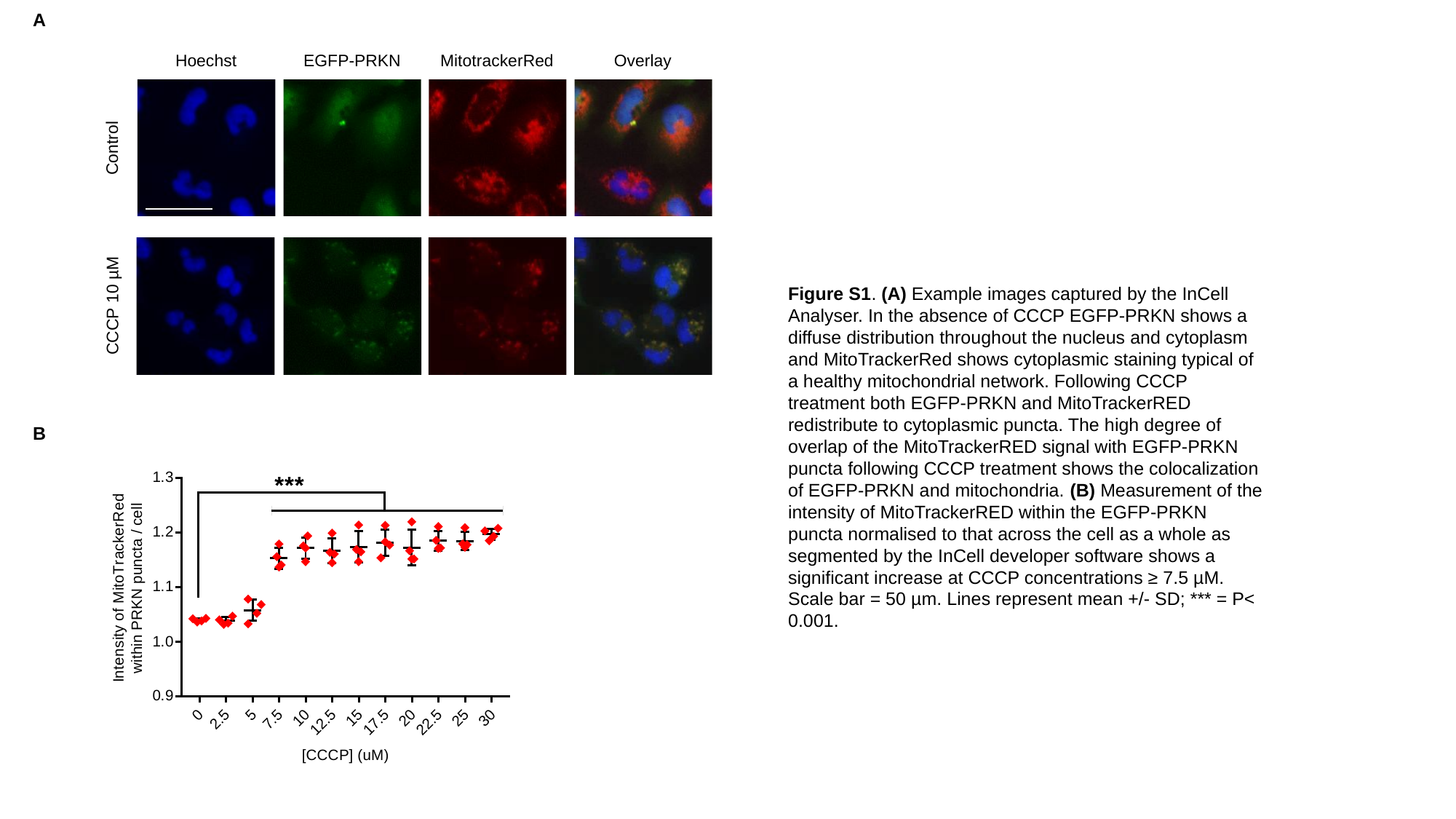

A
Hoechst
EGFP-PRKN
MitotrackerRed
Overlay
Control
Figure S1. (A) Example images captured by the InCell Analyser. In the absence of CCCP EGFP-PRKN shows a diffuse distribution throughout the nucleus and cytoplasm and MitoTrackerRed shows cytoplasmic staining typical of a healthy mitochondrial network. Following CCCP treatment both EGFP-PRKN and MitoTrackerRED redistribute to cytoplasmic puncta. The high degree of overlap of the MitoTrackerRED signal with EGFP-PRKN puncta following CCCP treatment shows the colocalization of EGFP-PRKN and mitochondria. (B) Measurement of the intensity of MitoTrackerRED within the EGFP-PRKN puncta normalised to that across the cell as a whole as segmented by the InCell developer software shows a significant increase at CCCP concentrations ≥ 7.5 µM. Scale bar = 50 µm. Lines represent mean +/- SD; *** = P< 0.001.
CCCP 10 µM
B

## Slide 2
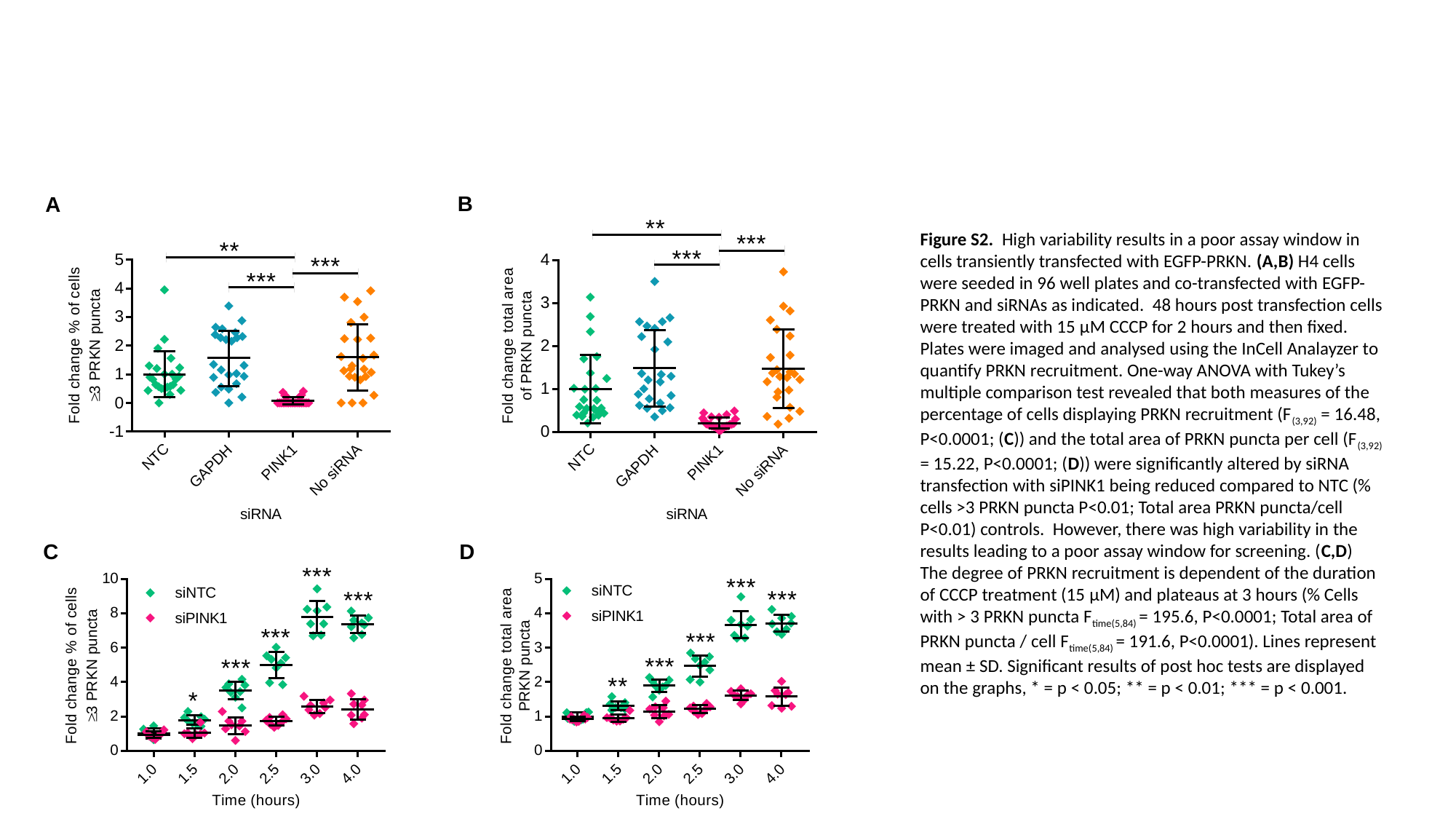

B
A
Figure S2. High variability results in a poor assay window in cells transiently transfected with EGFP-PRKN. (A,B) H4 cells were seeded in 96 well plates and co-transfected with EGFP-PRKN and siRNAs as indicated. 48 hours post transfection cells were treated with 15 μM CCCP for 2 hours and then fixed. Plates were imaged and analysed using the InCell Analayzer to quantify PRKN recruitment. One-way ANOVA with Tukey’s multiple comparison test revealed that both measures of the percentage of cells displaying PRKN recruitment (F(3,92) = 16.48, P<0.0001; (C)) and the total area of PRKN puncta per cell (F(3,92) = 15.22, P<0.0001; (D)) were significantly altered by siRNA transfection with siPINK1 being reduced compared to NTC (% cells >3 PRKN puncta P<0.01; Total area PRKN puncta/cell P<0.01) controls. However, there was high variability in the results leading to a poor assay window for screening. (C,D) The degree of PRKN recruitment is dependent of the duration of CCCP treatment (15 μM) and plateaus at 3 hours (% Cells with > 3 PRKN puncta Ftime(5,84) = 195.6, P<0.0001; Total area of PRKN puncta / cell Ftime(5,84) = 191.6, P<0.0001). Lines represent mean ± SD. Significant results of post hoc tests are displayed on the graphs, * = p < 0.05; ** = p < 0.01; *** = p < 0.001.
C
D

## Slide 3
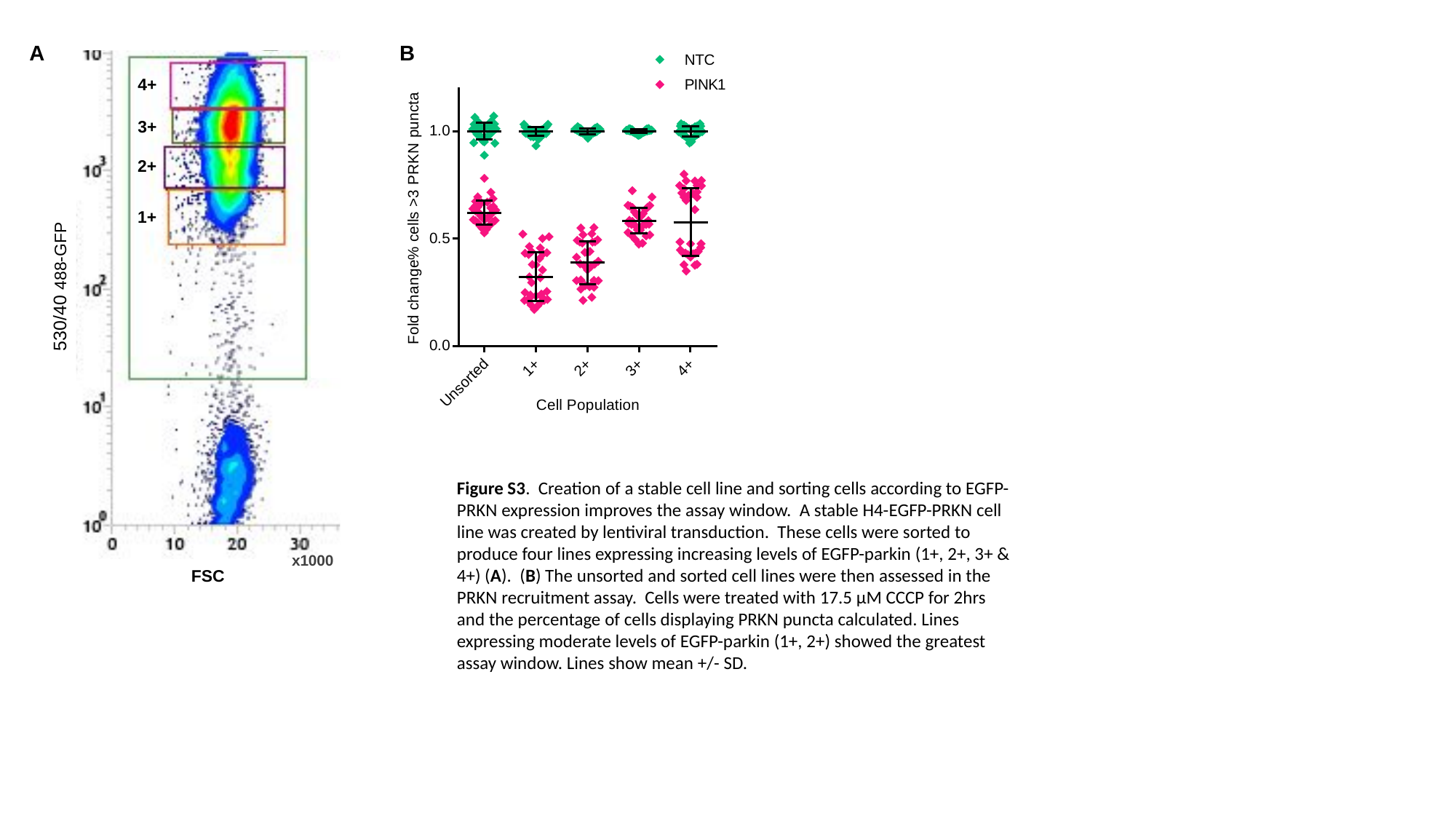

A
B
4+
3+
2+
1+
530/40 488-GFP
Figure S3. Creation of a stable cell line and sorting cells according to EGFP-PRKN expression improves the assay window. A stable H4-EGFP-PRKN cell line was created by lentiviral transduction. These cells were sorted to produce four lines expressing increasing levels of EGFP-parkin (1+, 2+, 3+ & 4+) (A). (B) The unsorted and sorted cell lines were then assessed in the PRKN recruitment assay. Cells were treated with 17.5 μM CCCP for 2hrs and the percentage of cells displaying PRKN puncta calculated. Lines expressing moderate levels of EGFP-parkin (1+, 2+) showed the greatest assay window. Lines show mean +/- SD.
x1000
FSC

## Slide 4
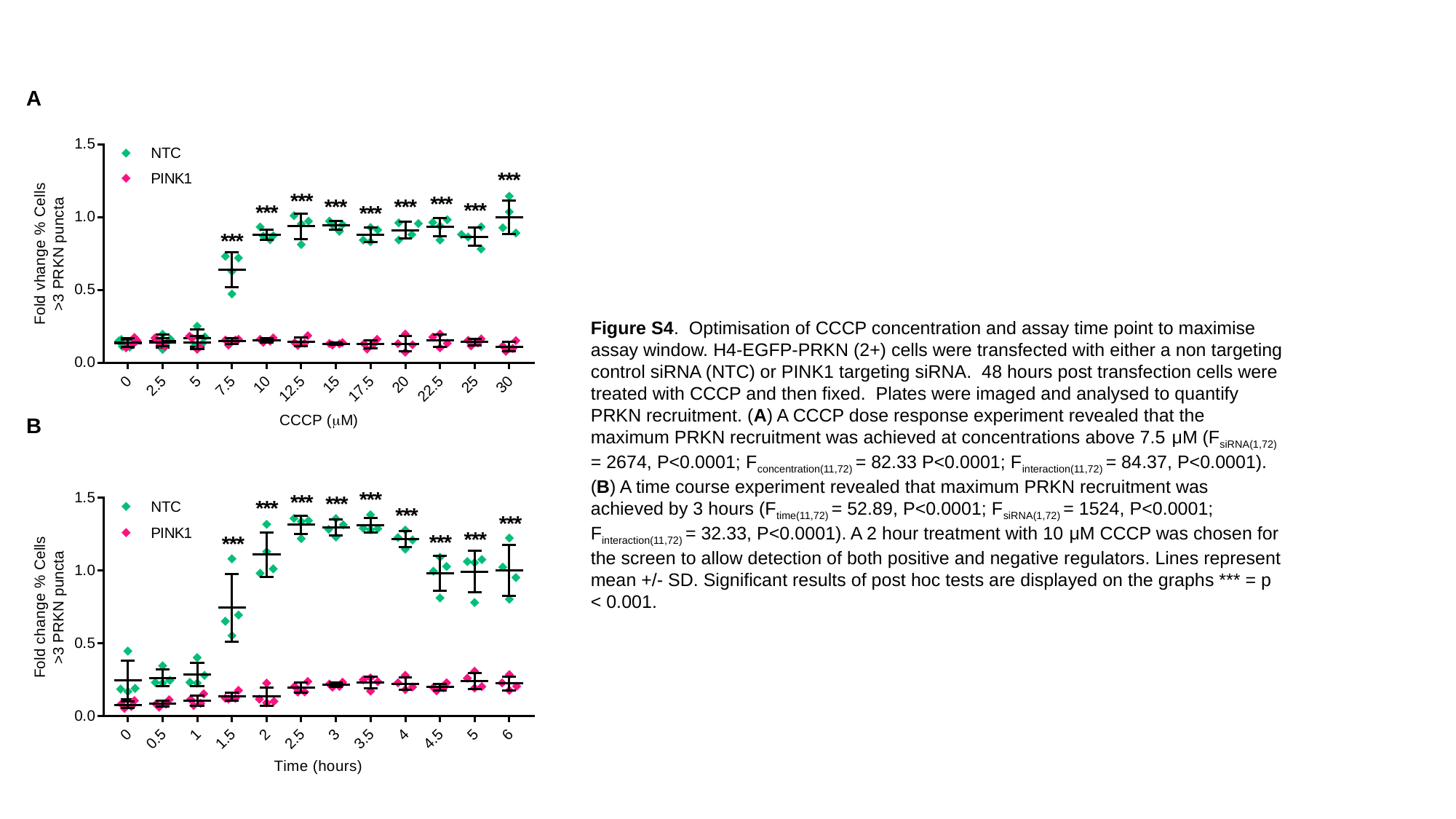

A
Figure S4.  Optimisation of CCCP concentration and assay time point to maximise assay window. H4-EGFP-PRKN (2+) cells were transfected with either a non targeting control siRNA (NTC) or PINK1 targeting siRNA.  48 hours post transfection cells were treated with CCCP and then fixed.  Plates were imaged and analysed to quantify PRKN recruitment. (A) A CCCP dose response experiment revealed that the maximum PRKN recruitment was achieved at concentrations above 7.5 μM (FsiRNA(1,72) = 2674, P<0.0001; Fconcentration(11,72) = 82.33 P<0.0001; Finteraction(11,72) = 84.37, P<0.0001). (B) A time course experiment revealed that maximum PRKN recruitment was achieved by 3 hours (Ftime(11,72) = 52.89, P<0.0001; FsiRNA(1,72) = 1524, P<0.0001; Finteraction(11,72) = 32.33, P<0.0001). A 2 hour treatment with 10 μM CCCP was chosen for the screen to allow detection of both positive and negative regulators. Lines represent mean +/- SD. Significant results of post hoc tests are displayed on the graphs *** = p < 0.001.
B

## Slide 5
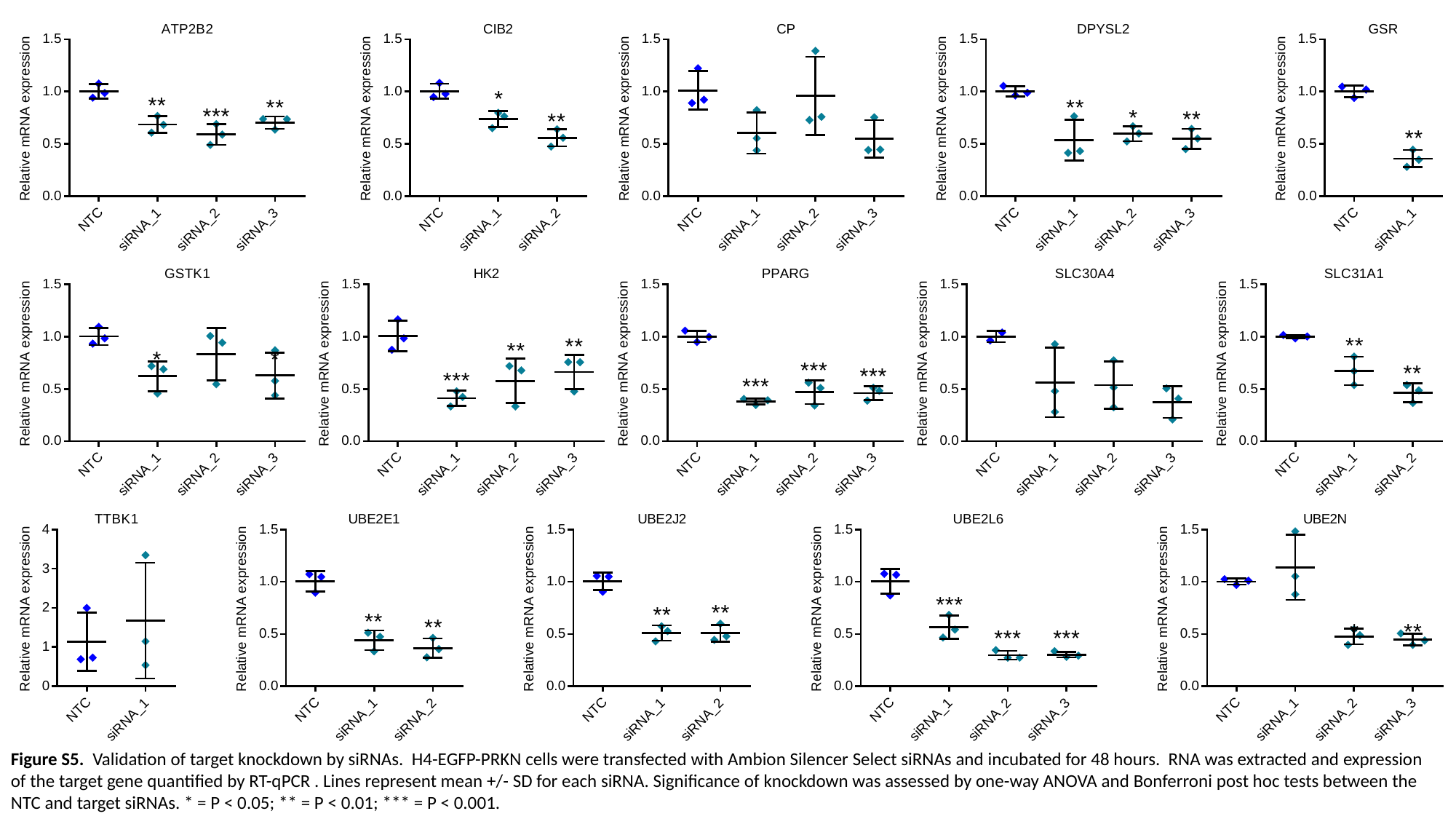

Figure S5. Validation of target knockdown by siRNAs. H4-EGFP-PRKN cells were transfected with Ambion Silencer Select siRNAs and incubated for 48 hours. RNA was extracted and expression of the target gene quantified by RT-qPCR . Lines represent mean +/- SD for each siRNA. Significance of knockdown was assessed by one-way ANOVA and Bonferroni post hoc tests between the NTC and target siRNAs. * = P < 0.05; ** = P < 0.01; *** = P < 0.001.

## Slide 6
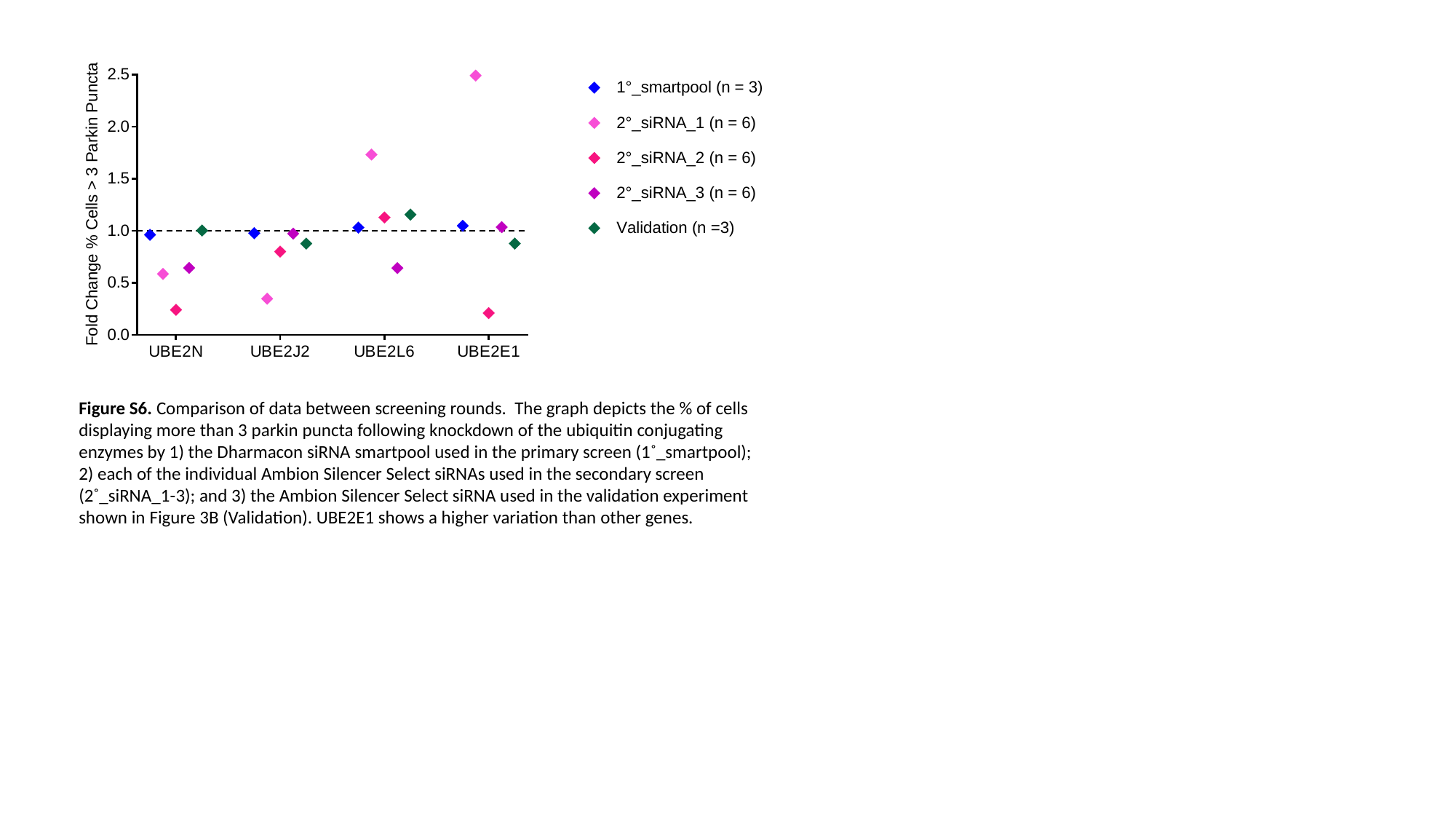

Figure S6. Comparison of data between screening rounds. The graph depicts the % of cells displaying more than 3 parkin puncta following knockdown of the ubiquitin conjugating enzymes by 1) the Dharmacon siRNA smartpool used in the primary screen (1˚_smartpool); 2) each of the individual Ambion Silencer Select siRNAs used in the secondary screen (2˚_siRNA_1-3); and 3) the Ambion Silencer Select siRNA used in the validation experiment shown in Figure 3B (Validation). UBE2E1 shows a higher variation than other genes.

## Slide 7
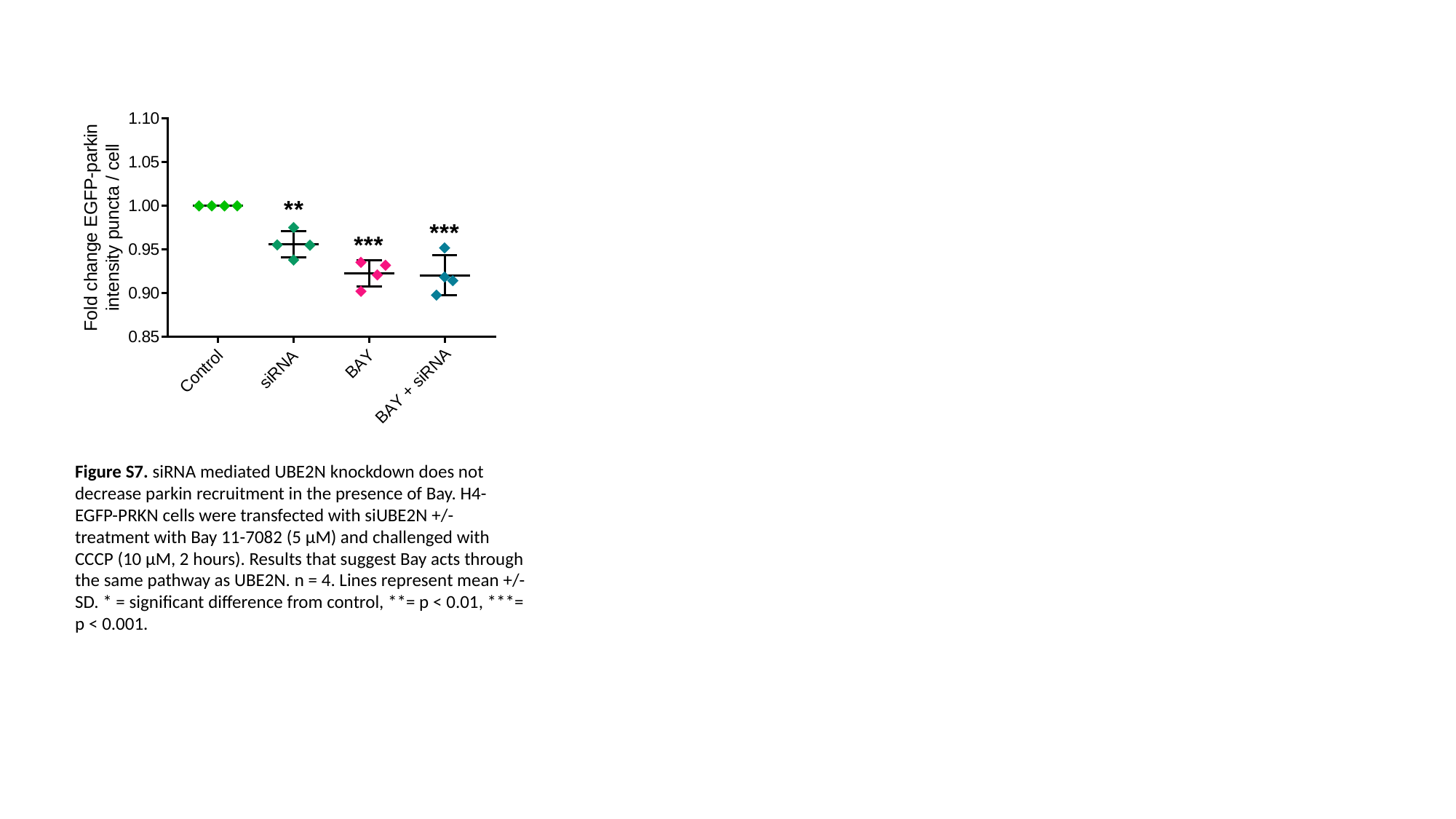

Figure S7. siRNA mediated UBE2N knockdown does not decrease parkin recruitment in the presence of Bay. H4-EGFP-PRKN cells were transfected with siUBE2N +/- treatment with Bay 11-7082 (5 μM) and challenged with CCCP (10 μM, 2 hours). Results that suggest Bay acts through the same pathway as UBE2N. n = 4. Lines represent mean +/- SD. * = significant difference from control, **= p < 0.01, ***= p < 0.001.

## Slide 8
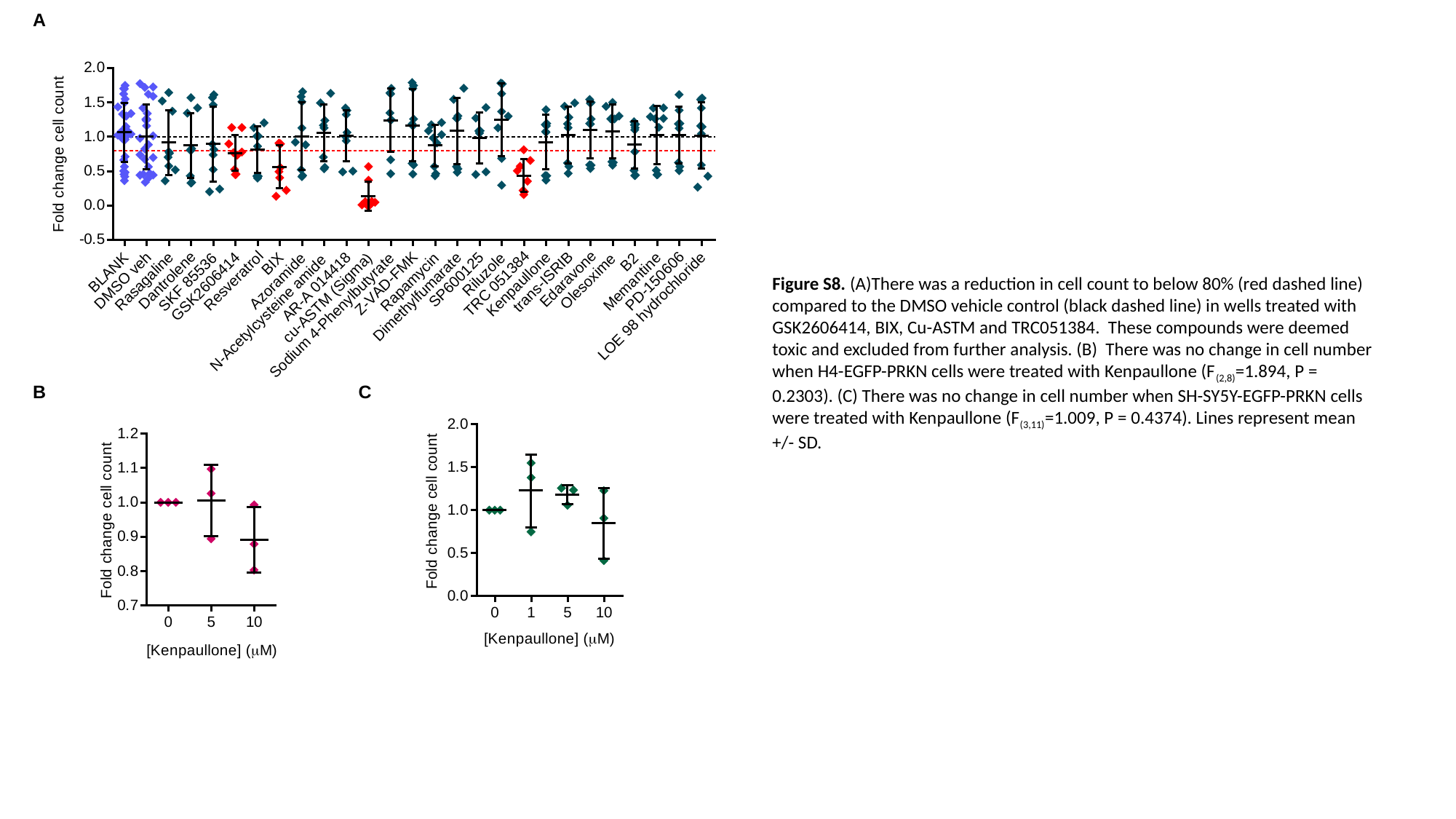

A
A
Figure S8. (A)There was a reduction in cell count to below 80% (red dashed line) compared to the DMSO vehicle control (black dashed line) in wells treated with GSK2606414, BIX, Cu-ASTM and TRC051384. These compounds were deemed toxic and excluded from further analysis. (B) There was no change in cell number when H4-EGFP-PRKN cells were treated with Kenpaullone (F(2,8)=1.894, P = 0.2303). (C) There was no change in cell number when SH-SY5Y-EGFP-PRKN cells were treated with Kenpaullone (F(3,11)=1.009, P = 0.4374). Lines represent mean +/- SD.
B
C

## Slide 9
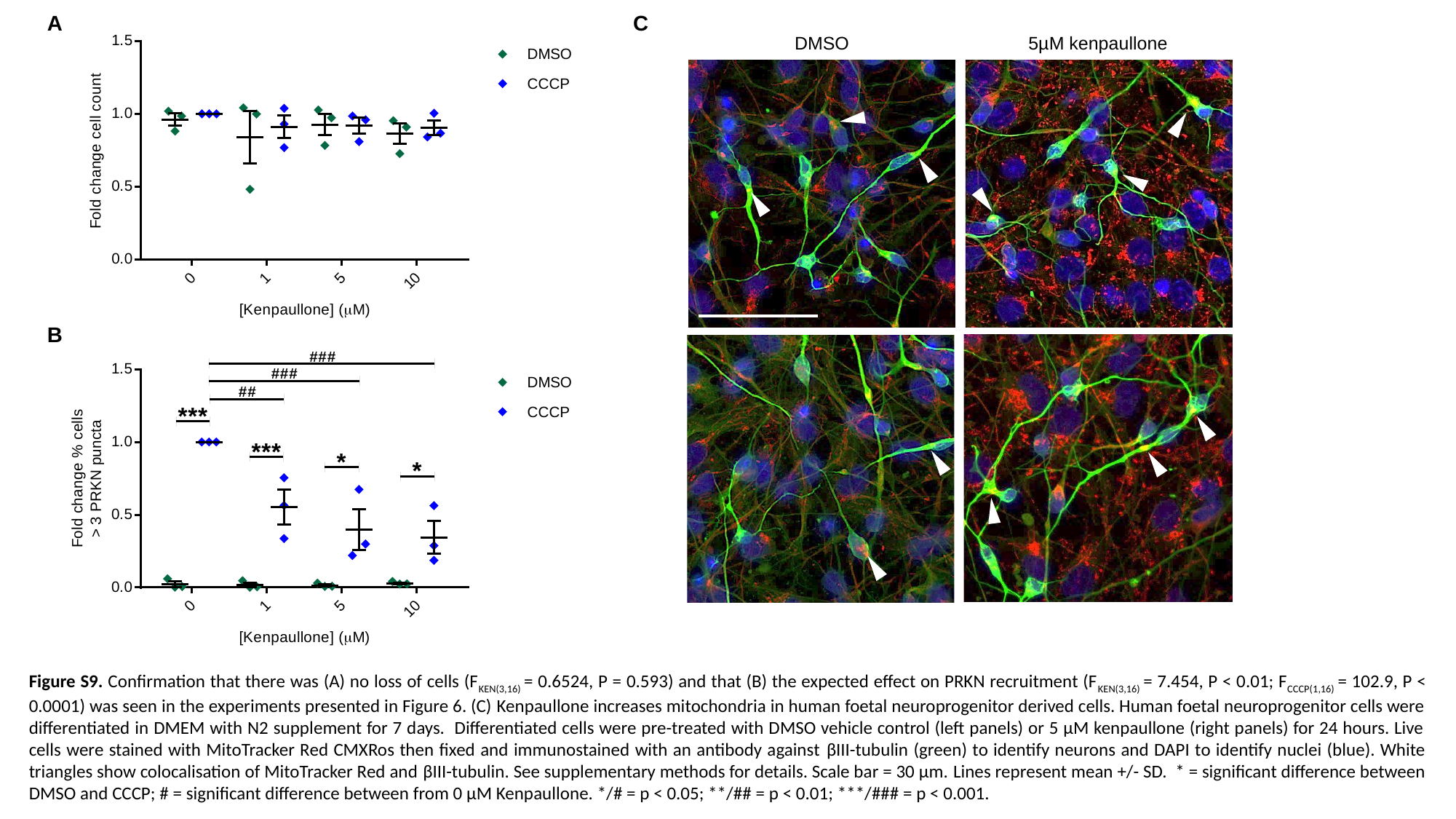

A
C
DMSO
5µM kenpaullone
B
Figure S9. Confirmation that there was (A) no loss of cells (FKEN(3,16) = 0.6524, P = 0.593) and that (B) the expected effect on PRKN recruitment (FKEN(3,16) = 7.454, P < 0.01; FCCCP(1,16) = 102.9, P < 0.0001) was seen in the experiments presented in Figure 6. (C) Kenpaullone increases mitochondria in human foetal neuroprogenitor derived cells. Human foetal neuroprogenitor cells were differentiated in DMEM with N2 supplement for 7 days. Differentiated cells were pre-treated with DMSO vehicle control (left panels) or 5 µM kenpaullone (right panels) for 24 hours. Live cells were stained with MitoTracker Red CMXRos then fixed and immunostained with an antibody against βIII-tubulin (green) to identify neurons and DAPI to identify nuclei (blue). White triangles show colocalisation of MitoTracker Red and βIII-tubulin. See supplementary methods for details. Scale bar = 30 µm. Lines represent mean +/- SD. * = significant difference between DMSO and CCCP; # = significant difference between from 0 µM Kenpaullone. */# = p < 0.05; **/## = p < 0.01; ***/### = p < 0.001.

## Slide 10
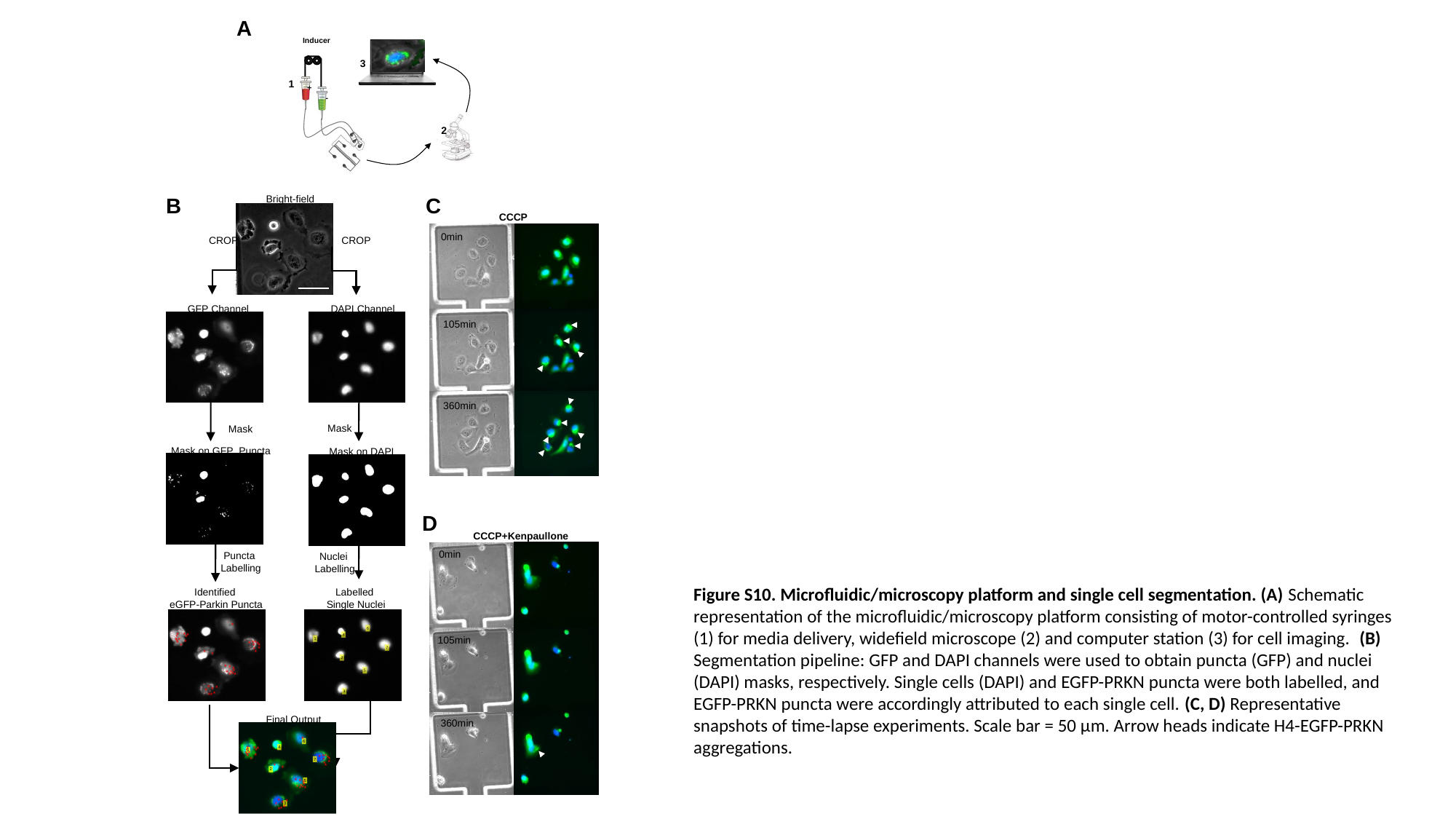

A
Inducer
3
1
2
+
-
B
C
Bright-field
CROP
CROP
GFP Channel
DAPI Channel
Mask
Mask
 Mask on GFP_Puncta
Mask on DAPI
Puncta
Labelling
Nuclei
Labelling
Identified
eGFP-Parkin Puncta
Labelled
Single Nuclei
*
*
*
*
*
*
*
*
*
*
*
*
*
*
*
*
*
*
*
*
*
*
*
*
*
*
*
*
*
*
*
*
*
*
*
*
*
*
*
Final Output
6
4
1
7
2
5
3
*
*
*
*
*
*
*
*
*
*
*
*
*
*
*
*
*
*
*
*
*
*
*
*
*
*
*
*
*
*
*
*
*
*
*
*
*
*
*
CCCP
0min
105min
360min
D
CCCP+Kenpaullone
0min
105min
360min
Figure S10. Microfluidic/microscopy platform and single cell segmentation. (A) Schematic representation of the microfluidic/microscopy platform consisting of motor-controlled syringes (1) for media delivery, widefield microscope (2) and computer station (3) for cell imaging. (B) Segmentation pipeline: GFP and DAPI channels were used to obtain puncta (GFP) and nuclei (DAPI) masks, respectively. Single cells (DAPI) and EGFP-PRKN puncta were both labelled, and EGFP-PRKN puncta were accordingly attributed to each single cell. (C, D) Representative snapshots of time-lapse experiments. Scale bar = 50 µm. Arrow heads indicate H4-EGFP-PRKN aggregations.

## Slide 11
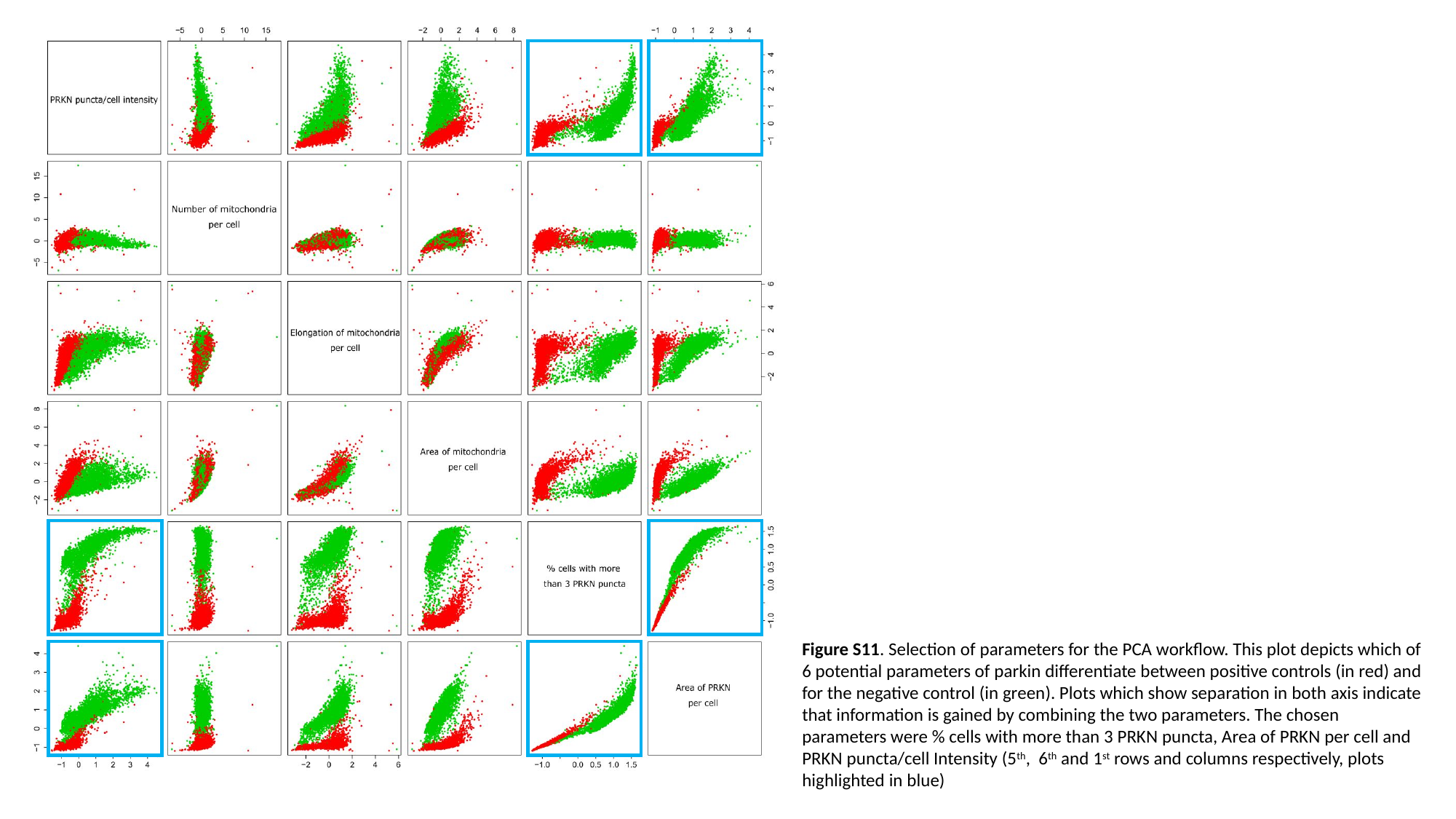

Figure S11. Selection of parameters for the PCA workflow. This plot depicts which of 6 potential parameters of parkin differentiate between positive controls (in red) and for the negative control (in green). Plots which show separation in both axis indicate that information is gained by combining the two parameters. The chosen parameters were % cells with more than 3 PRKN puncta, Area of PRKN per cell and PRKN puncta/cell Intensity (5th, 6th and 1st rows and columns respectively, plots highlighted in blue)

## Slide 12
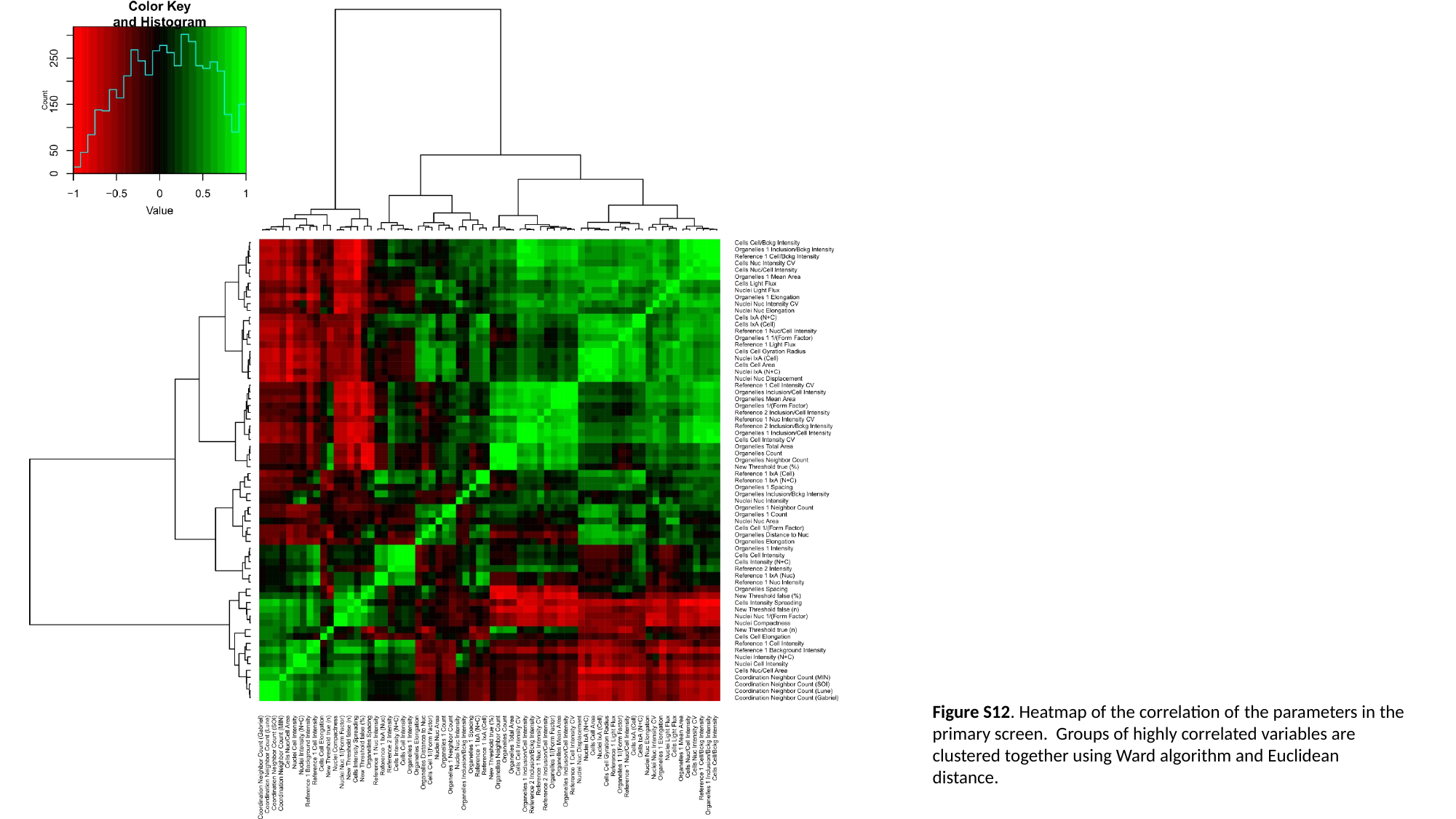

Figure S12. Heatmap of the correlation of the parameters in the primary screen. Groups of highly correlated variables are clustered together using Ward algorithm and Euclidean distance.

## Slide 13
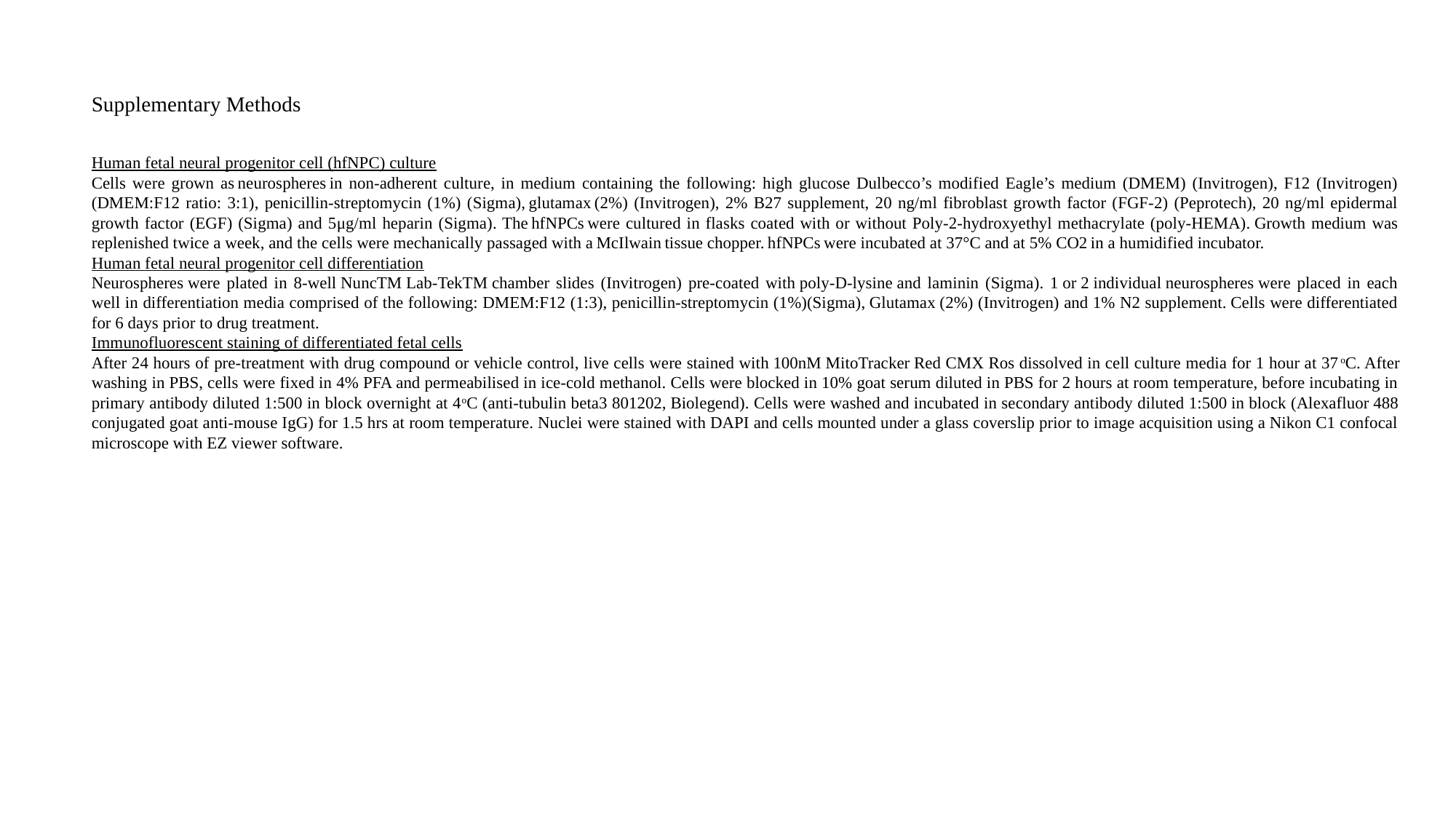

Supplementary Methods
Human fetal neural progenitor cell (hfNPC) culture
Cells were grown as neurospheres in non-adherent culture, in medium containing the following: high glucose Dulbecco’s modified Eagle’s medium (DMEM) (Invitrogen), F12 (Invitrogen) (DMEM:F12 ratio: 3:1), penicillin-streptomycin (1%) (Sigma), glutamax (2%) (Invitrogen), 2% B27 supplement, 20 ng/ml fibroblast growth factor (FGF-2) (Peprotech), 20 ng/ml epidermal growth factor (EGF) (Sigma) and 5μg/ml heparin (Sigma). The hfNPCs were cultured in flasks coated with or without Poly-2-hydroxyethyl methacrylate (poly-HEMA). Growth medium was replenished twice a week, and the cells were mechanically passaged with a McIlwain tissue chopper. hfNPCs were incubated at 37°C and at 5% CO2 in a humidified incubator.
Human fetal neural progenitor cell differentiation
Neurospheres were plated in 8-well NuncTM Lab-TekTM chamber slides (Invitrogen) pre-coated with poly-D-lysine and laminin (Sigma). 1 or 2 individual neurospheres were placed in each well in differentiation media comprised of the following: DMEM:F12 (1:3), penicillin-streptomycin (1%)(Sigma), Glutamax (2%) (Invitrogen) and 1% N2 supplement. Cells were differentiated for 6 days prior to drug treatment.
Immunofluorescent staining of differentiated fetal cells
After 24 hours of pre-treatment with drug compound or vehicle control, live cells were stained with 100nM MitoTracker Red CMX Ros dissolved in cell culture media for 1 hour at 37oC. After washing in PBS, cells were fixed in 4% PFA and permeabilised in ice-cold methanol. Cells were blocked in 10% goat serum diluted in PBS for 2 hours at room temperature, before incubating in primary antibody diluted 1:500 in block overnight at 4oC (anti-tubulin beta3 801202, Biolegend). Cells were washed and incubated in secondary antibody diluted 1:500 in block (Alexafluor 488 conjugated goat anti-mouse IgG) for 1.5 hrs at room temperature. Nuclei were stained with DAPI and cells mounted under a glass coverslip prior to image acquisition using a Nikon C1 confocal microscope with EZ viewer software.
